# Huanglongbing impairs the rhizosphere-to-rhizoplane enrichment process of the citrus root-associated microbiome

**DOI:** 10.1186/s40168-017-0304-4

**Published:** 2017-08-10

**Authors:** Yunzeng Zhang, Jin Xu, Nadia Riera, Tao Jin, Jinyun Li, Nian Wang

**Affiliations:** 1 Citrus Research and Education Center, Department of Microbiology and Cell Science, IFAS, University of Florida, Lake Alfred, FL USA; 2 BGI-Shenzhen, Shenzhen, China; 3 China-USA Citrus Huanglongbing Joint Laboratory (A joint laboratory of The University of Florida’s Institute of Food and Agricultural Sciences and Gannan Normal University), National Navel Orange Engineering Research Center, Gannan Normal University, Ganzhou, Jiangxi China

**Keywords:** Rhizoplane microbiome, Rhizosphere microbiome, Huanglongbing, Metagenome, Metatranscriptome, Liberibacter

## Abstract

**Background:**

Roots are the primary site for plant-microbe interactions. Among the three root-associated layers (i.e., rhizosphere, rhizoplane, and endorhiza), the rhizoplane is a key component serving a critical gating role that controls microbial entry into plant roots. The microbial communities colonizing the three layers are believed to be gradually enriched from the bulk soil inoculum. However, it is unknown how this enrichment process, particularly the rhizosphere to rhizoplane step, is affected by biotic stresses, such as disease. In this study, we address this question using the citrus root-associated microbiome as a model.

**Results:**

We identified the rhizosphere-to-rhizoplane-enriched taxonomic and functional properties of the citrus root-associated microbiome and determined how they were affected by Huanglongbing (HLB), a severe systemic disease caused by *Candidatus* Liberibacter asiaticus, using metagenomic and metatranscriptomic approaches. Multiple rhizoplane-enriched genera were identified, with *Bradyrhizobium* and *Burkholderia* being the most dominant. Plant-derived carbon sources are an important driving force for the enrichment process. The enrichment of functional attributes, such as motility, chemotaxis, secretion systems, and lipopolysaccharide (LPS) synthesis, demonstrated more active microbe-plant interactions on the rhizoplane than the rhizosphere. We observed that HLB impaired the rhizosphere-to-rhizoplane enrichment process of the citrus root-associated microbiome in three ways: (1) by decreasing the relative abundance of most rhizoplane-enriched genera; (2) by reducing the relative abundance and/or expression activity of the functional attributes involved in microbe-plant interactions; and (3) by recruiting more functional features involved in autotrophic life cycle adaptation, such as carbon fixation and nitrogen nitrification in the HLB rhizoplane microbiome. Finally, our data showed that inoculation of *Burkholderia* strains isolated from the healthy citrus root-associated microbiome could trigger the expression of genes involved in induced systemic resistance in inoculated plants.

**Conclusions:**

HLB causes decreased relative abundance and/or expression activity of rhizoplane-enriched taxonomic and functional properties, collectively resulting in impaired plant host-microbiome interactions. Manipulation of the citrus root-associated microbiome, for instance, by inoculating citrus roots with beneficial *Burkholderia* strains, has potential to promote plant health. Our results provide novel insights for understanding the contributions of the community enrichment process of the root-associated microbiome to the plant hosts.

**Electronic supplementary material:**

The online version of this article (doi:10.1186/s40168-017-0304-4) contains supplementary material, which is available to authorized users.

## Background

Plants harbor a high diversity of microorganisms proximal to, on, and inside their tissues. These microorganisms, which mostly include bacteria, Archaea, and fungi, form microbial communities that are associated with various plant habitats, including the rhizosphere, rhizoplane, phyllosphere, and endosphere, and the communities are collectively known as plant microbiomes [1–3]. The plant microbiome can determine the fate of plants in multiple ways, including (i) supplying plants with nutrition, e.g., nitrogen fixation or phosphate solubilization; (ii) modulating plant growth or relieving stress through phytohormone production or degradation; (iii) maintaining plant health through competition with pathogens or induction of plant resistance [4–7]; and (iv) driving the evolution of multi-disease resistance during long-term coevolution history [8]. Given its importance, the microbiome is considered an integral component of the plant-host assemblage, which is recognized as a “holobiont” [4]. Understanding the plant microbiome will have important implications for the human food supply and its security, biodiversity, and ecosystem functionality [9, 10].

The plant microbiome is predominantly assembled from external inoculum pools, including soil- and air-borne microbial communities [6]. Soil is the largest known reservoir of microbial diversity [11], and roots are the primary site for plant-microbe interactions. Recently, several studies were performed to reveal the assembly mechanisms of the plant root-associated microbiome [12–17]. Based on these studies, a three-step enrichment model is proposed for the root-associated microbiome assembly process. Briefly, a fraction of microorganisms in the bulk soil inoculum pool are enriched toward the roots by the general gradients of carbon source, phytochemicals, pH, oxygen, and nutrients imposed by the roots, and these form the rhizosphere microbiome. Secondly, a more specialized community is further enriched on the rhizoplane; more intimate microbe-host interactions occur and much stronger selection pressure from the plant host is applied at the assembled rhizoplane microbiome than the rhizosphere microbiome. Finally, certain microorganisms enter and inhabit the endorhiza, and the established endorhiza microbiome contributes significantly to plant fitness, including immune system modulation [5]. Therefore, among the three root-associated layers described above, the rhizoplane is a key component serving a critical gating role that controls microbial entry into plant roots. However, these previous studies were mainly dedicated to revealing the effects of the plant genomic background on the microbial community assembly process [12–17]; how the enrichment process of the microbiome is affected by biotic stresses such as disease remains elusive. In addition, the studies were conducted using annual plants including *Arabidopsis*, soybean, barley, wheat, cucumber, and rice. Compared to the short lifespan of annual plants, perennial plants are subjected to longer periods of plant-microbe interactions for a single generation, including complicated root growth patterns and variable environmental factors. Compared to annual plants, the microbiome assembly cues of perennial plants are less studied. Here, we investigated the microbial community assembly process of the root-associated microbiome of citrus, a perennial plant, and how Huanglongbing (HLB) affects the microbiome enrichment process.

HLB is the most devastating citrus disease worldwide [18, 19]. The disease is caused by the gram-negative, phloem-limited, alpha-proteobacteria *Candidatus* Liberibacter spp., i.e., Ca. *L. asiaticus* (Las), Ca. *L. africanus* (Laf), and Ca. *L. americanus* (Lam) [18, 19]. HLB impairs phloem transportation of photoassimilates [18] and causes root decline [20]. Las living in the phloem prevents direct interactions with other microbes on the rhizoplane and in the rhizosphere. Instead, Las causes decreased photoassimilate transportation, likely reducing plant resource availability for the root-associated microbiome. Our previous results suggested that HLB significantly altered the structure or functional potential of the citrus endosphere (leaves or roots) or rhizosphere bacterial community based on cultivation, 16S rDNA clone library, PhyloChip, or the GeoChip method [21–24]. However, the low-throughput-based methods used in these previous studies limited our understanding of the citrus microbiome. Additionally, these studies focused on the bacterial community colonized on a single layer, either the rhizosphere or endosphere. How HLB affects the microbiome assembly process, particularly the process from the less tightly associated rhizosphere to the rhizoplane component, of the citrus root-associated microbiome remains unknown. In this study, we obtained snapshots of the citrus root microbiome using metagenomic (MG) and metatranscriptomic (MT) approaches to investigate the rhizosphere-to-rhizoplane-enriched taxa and functions and how HLB affects those enriched taxonomic and functional attributes.

## Methods

### Sample collection

Three healthy and three Las-infected 11-year-old “midsweet” sweet orange on Swingle citrumelo rootstock from a grove at Auburndale (28.11 N, 81.79 W), Florida, USA were identified based on visual symptoms and qPCR results [24] from both leaf and root samples (Additional file 1: Figure S1). The fine root cores (i.e., roots with approximately 2-cm-thick adjacent soil layers) from four corners of each tree were collected for DNA and RNA extraction. The loosely attached soil on the roots was removed with gentle shaking. The rhizosphere soil was carefully and quickly collected by gently brushing the remaining soil adhering to the roots using brush pencils. The soil collection step was performed on ice. Then, the roots were placed in pre-cooled PBS (phosphate-buffered saline) buffer, and the rhizoplane soil was extracted by ultra-sonication as described by Edwards et al. [17]. The roots were sonicated twice for 20 s each (time interval 5 s) using a sonication bath (power 130 W, 60 Hz, Fisher Scientific). The roots were discarded, and the rhizoplane soil was collected by centrifugation at 12,000×*g* for 1 min at 4 °C. Each rhizosphere or rhizoplane soil sample was re-suspended in LifeGuard soil preservation solution (Mobio Laboratories) immediately after processing. The full procedure was performed sample by sample and as quickly as possible (three persons worked together, total processing time was <5 min for each sample). The soil samples from the four corners of each tree were pooled into a single sample. The samples were stored at −20 °C until further processing.

### Nucleic acid extraction and sequencing

DNA and RNA were extracted from 2 g of each soil sample using a RNA PowerSoil total RNA isolation kit and the RNA PowerSoil DNA Elution Accessory Kit following the manufacturer’s protocol (MO BIO Laboratories, Inc.). In this extraction process, DNA and RNA were extracted from the same sample simultaneously (i.e., DNA and RNA were eluted from the same column with different elution buffer), and thus, the RNA samples reflect the real-time gene expression profiling of the corresponding DNA samples. Large-scale shotgun metagenome and metatranscriptome sequencing were performed on the Illumina Hiseq4000 platform by Novogene (Novogene, Beijing, China). Briefly, the DNA samples were randomly sheared using Covaris Ultrasonic Processor into approximately 300 bp fragments, which were then used to construct the sequencing libraries using the Illumina TruSeq® DNA PCR-free sample preparation kit (Illumina, USA). For the RNA samples, the ribosomal RNA was depleted using the Ribo-Zero™ rRNA Removal Kit for Bacteria (Illumina, Madison, USA) according to the manufacturer’s instructions. The remaining transcripts were fragmented and reverse-transcribed. The messenger RNA (mRNA) libraries were prepared using the TrueSeq Stranded mRNA Sample Prep kit (Illumina, USA), and 2 × 125-bp paired-end reads were generated for all samples. Approximately 10 Gb sequencing data for each DNA shotgun sequencing sample and 8 Gb data for each RNA sample were obtained. The DNA and RNA reads were deposited at NCBI under the bioproject accession no. PRJNA324090 and SRA accession no. SRP076109.

### Bioinformatics analyses

The raw reads from metagenome sequencing were filtered, trimmed, and quality-controlled to generate the clean reads, which were further trimmed using Sickle [25] with the parameters –q 20 and –l 80. On average, 4.85% of the clean reads were discarded from this trimming step. The trimmed reads were aligned to the Swingle citrumelo genome [26], sweet orange genome [27], and *Citrus clementina* genome [28] using bowtie2 [29], and the corresponding mapped reads were removed. Only the reads that did not map to any of the three citrus genomes were retained for further analysis.

The filtered reads from all 12 samples were pooled and subjected to de novo assembly using megahit v1.03 [30]. The metagenes were predicted using MetaGeneMark [31]. The non-redundant gene categories (unigenes) were generated using CD-HIT-est with an identity cutoff of 95% [32]. To obtain the taxonomic annotation for the unigenes, the protein sequences were aligned against the NCBI microbial NR database using DIAMOND software [33] with an E value cutoff of 1e-5. Then, the taxonomic annotations were assigned using the MEGAN LCA annotation method [34]. The functional annotation was assigned to the unigenes by blasting against the KEGG orthology database using DIAMOND software.

To generate taxonomic and functional abundance and expression profiling, the short DNA and RNA reads from each sample were aligned to the unigenes using SOAP2 [35] with default parameters. The generated alignments were parsed, and abundance and expression profiling were obtained (reads count matrixes). Based on the abundance and expression profiles, the features (genera and KOs) with significantly differential abundance or expression activity were determined using DESeq2 with a negative binomial generalized linear model (*p* < 0.05) [36]. For the rhizoplane-enriched feature detection, paired DESeq2 comparison analysis was performed separately for healthy samples and HLB samples to prevent the potential effects of health status on the results, and the identified features were merged. Other comparisons, such as healthy vs. HLB samples for differential rhizoplane taxonomic and functional feature determination, were conducted using DESeq2, where the individual samples from the healthy or HLB trees were treated as biological replicates. The taxonomic and functional dissimilarity analyses among samples were performed using the *R* package VEGAN [37] with a Bray-Curtis distance matrix. The variation partitioning analysis (VPA) was performed based on the taxonomic and functional composition matrix calculated on the genus and KEGG orthologue (KO) level of all 12 samples (rhizosphere and rhizoplane, healthy and HLB) using VEGAN. The heatmap and Venn diagram plots were drawn using the gplots and VennDiagram package [38, 39], respectively. The average genome size (AGS) for the metagenomic samples was estimated using MicrobeCensus v1.0.7 based on 10 million reads [40]. The bacterial secretion system-based effector genes were identified by blasting the unigenes against a custom-built type III, IV, and VI effector library (T3SE: http://effectors.bic.nus.edu.sg/T3SEdb/index.php; T4SE: http://sate.cirad.fr/; T6SE: http://db-mml.sjtu.edu.cn/SecReT6/) with an amino acid identity cutoff of 45% and a coverage of 80% [41]. To explore the relative contribution of taxa to the rhizosphere to rhizoplane-enriched KOs, the taxonomic information for each selected gene (at genus and family level) was extracted, and their relationships were calculated using the method described by Ofek-Lalzar et al. [15]. The metagenome-assembled genome (MAG) extraction was performed using MetaBAT [42], and the quality of the generated MAGs was checked using CheckM [43]. The genome similarity between selected MAGs and their related genomes was determined using the GGDC2.1 server [44]. The MAGs were annotated using the RAST server and blast2GO software [45, 46]. The annotated bin.74 and bin.105 were deposited in the RAST server with the job id 311713 and 312590, respectively.

### *Burkholderia* sp. isolation and survival assay

Bacterial strains were isolated from the rhizosphere of healthy citrus plants in a relevant study (Riera et al., Unpublished). Based on the 16S rDNA gene sequencing results using the universal primers 27F and 1492R [47], the strains were identified. *Burkholderia* strains were selected for the antagonistic activity test against several known citrus pathogens. Two representative *Burkholderia* strains, namely, *Burkholderia metallica* (strain A53) and *Burkholderia territori* (strain A63), which showed the best antagonistic activities among the tested strains, were selected for further analysis.

The two strains were transformed with the pUFR034-gfp plasmid by electroporation. Positive GFP (green fluorescent protein)-labeled strains were verified by fluorescent microscopy and qPCR results. Each transformed *Burkholderia* strain was applied by soil drench (15 mL of 1 × 10^8^ cfu/mL) to five 1-year-old potted “Valencia” sweet orange plants grown in a quarantine greenhouse facility at the Citrus Research and Education Center, University of Florida, in Florida, USA, with controlled temperature (28–35 °C) and a relative humidity of 80%. The rhizosphere and rhizoplane soil samples were collected as described above (but without the LifeGuard soil preservation solution resuspension step) 1 h after inoculation (0 days post inoculation (dpi)), 2, 5, and 9 dpi. The genomic DNA was extracted using a PowerSoil DNA Isolation Kit (MO BIO Laboratories, Inc.). The population dynamics of the inoculated strains inside the rhizosphere and rhizoplane bacterial community (calculated as relative abundance change compared to the 0 dpi data) were calculated [48], with the total bacteria population serving as the reference. A primer pair targeting the GFP gene (GFPF 5′-TCCATGCCATGTGTAATCCC-3′, GFPR 5′-CCATTACCTGTCCACACAATCT-3′) was used for the detection of the inoculated strains. qPCR assays were conducted on a 7500 Fast Real-Time PCR System (Applied Biosystems, Foster City, CA, USA) using a Quantifast® SYBER® Green PCR kit (QIAGEN) following the manufacturer’s instructions with the following cycling conditions: an initial denaturation step of 5 min at 95 °C and 35 cycles of 10 s at 95 °C and 30 s at 60 °C. The primer set Eub338 and Eub518, which targets the conserved region of the 16S rRNA gene, was used for the detection of total bacteria, and qPCR assays were performed as described by Fierer et al. [49]. Furthermore, the long-term survival rate of the strains was also determined using 2-year-old Duncan grapefruit plants with three plants/replicates grown under the same growth conditions described above. One gram of rhizosphere soil from each tree was collected, and colony-forming units (CFU) were calculated using nutrient agar plates supplemented with 50 μg/ml kanamycin (strain native resistance for both strains). The CFU formation was determined at 0, 46, 60, 72, 91, 110, 131, 150, and 224 dpi.

### Expression of plant defense-related genes in response to beneficial bacteria treatment

Fifteen milliliters of 1 × 10^8^ cfu/mL of strains A53 and A63 were inoculated onto 1-year-old Valencia plants. Application of 15 mL of acibenzolar-S-methyl (ASM) (active ingredient in Actigard 50WG)  at 0.33 mg/ml was used as a positive control. The active ingredient of Actigard is an analogue of salicylic acid and systemic elicitor of plant defense [50]. A water only inoculation was used as a negative control. For each treatment, nine seedlings were used, with each seedling as a replicate.

To determine whether the treatments induced plant defense, we measured the expression of three defense-related genes: SAM, encoding *S*-adenosyl-l-methionine-salicylic acid carboxyl methyltransferase; PR1, encoding pathogenesis-related protein 1; and PR2, encoding pathogenesis-related protein 2 [50] using quantitative reverse transcription PCR (qRT-PCR). The primer sequences for *SAM* and *PR1* are SAMF 5′-GGACGCATCTTCTTGGGATAA-3′/SAMR 5′-CGTGACAGTTTCCTTGACGA-3′ and PR1F 3′-CAGGGTCTCCAAGCAACTATG-5′/PR1R 5′-CCACCTCGCGTATTTCTCTAA-3′, respectively. The primer sequences for PR2 were described previously [50]. Gene expression was determined at 0, 3, 5, and 7 dpi. For each time point, three biological repeats were sampled by collecting one leaf from three different plants. RNA was extracted using an RNeasy Mini Kit (QIAGEN) following the manufacturer’s instructions. Samples were treated with Ambion® DNA-free DNase Treatment and Removal Reagents. The qRT-PCR was performed using a Verso 1-step RT-qPCR Kit (ThermoFisher), and the fold change was calculated using the ∆∆Ct method as previously described [48].

## Results

### Structure and function of citrus root-associated microbiome

We collected the rhizosphere and rhizoplane soil samples from three healthy and three HLB-diseased citrus trees for MG and MT analyses. We generated 515,129,383 paired-end clean reads, which is equal to 129 Gb for MG, and 413,225,469 paired-end clean reads, which is equal to 103 Gb for MT, for the 12 samples. The citrus host-originated MG reads were depleted by aligning the reads to the three available citrus draft genomes to comprehensively remove the host-originated reads, and 0.039 to 1.79% clean reads were removed (Additional file 1: Table S1). We pooled the MG reads from all 12 samples in total of 501,171,627 paired-end reads (approximately 120 Gb) for de novo assembly and obtained an assembly of 10.84 Gb across 17,676,569 contigs, with the longest contig at 536,098 bp and N50 at 651 bp (based on all contigs ≥200 bp) (Additional file 1: Table S2). In total, 22,192,564 putative protein-coding genes were predicted from the assembly. After removing redundant sequences (identity >95%), 21,380,400 unigenes were generated. The unigenes represented more rhizoplane reads (46.48 ± 4.98%, mean ± SD, same herein) than rhizosphere reads (35.44 ± 2.34%) (*p* = 0.0006).

Taxonomy annotation was successfully assigned to 70% of the total unigenes. Bacteria comprised the predominant domain (99.30 ± 0.37%, mean relative abundance ± SD, *n* = 12), with small fractions of Archaea, eukaryotes, and viruses detected based on the annotated unigenes. *Proteobacteria* (74.56 ± 8.59%), *Actinobacteria* (16.80 ± 6.95%), *Bacteroidetes* (2.86 ± 0.97%), and *Acidobacteria* (2.42 ± 0.69%) were the dominant phyla (relative abundance ≥1%) (Additional file 1: Figure S2). Thaumarchaeota was the dominant phylum in the Archaea domain (0.41 ± 0.31%), and Ascomycota represented the most abundant phylum affiliated with fungi (0.14 ± 0.06%). Using Diamond BLASTP against the KEGG KO database, 53% of the unigenes were assigned KO function annotation, with most of the KO-annotated genes (94.3%) mapped to the KEGG pathways. In total, 8816 KOs were identified from the unigenes. KOs involved in amino acid metabolism (9.56 ± 0.06%), carbohydrate metabolism (8.65 ± 0.09%), membrane transport (8.51 ± 0.37%), and energy metabolism (5.67 ± 0.10%) were dominant (relative abundance ≥5%) based on KEGG level 2 pathway annotations.

Approximately 3.05 ± 1.39% of the metatranscriptomic reads were identified as rRNA, as derived by the SortMeRNA ver. 2.1 program [51], indicating a high-efficiency rRNA depletion strategy during the MT library construction. In addition, 32.94 ± 8.36% of the RNA clean reads from the rhizoplane samples and 27.38 ± 4.91% from the rhizosphere samples could be mapped to the unigenes. On average, 24.26% (ranging from 18.01 to 41.14%) of the unigenes identified in each sample were actively expressed (Additional file 1: Table S3). The community composition results from the MG and MT analyses revealed a similar structure when tested at the genus level (correlation coefficient *r* = 0.90, *p* < 0.005).

### Effects of HLB on the rhizosphere- to rhizoplane-enriched taxa

The alpha diversity (Shannon index) of the rhizosphere samples was significantly increased compared to the rhizoplane samples at low (e.g., phylum) or high (e.g., genus) resolution (Additional file 1: Table S4). The AGS of the rhizoplane microbiome was slightly but significantly increased compared to the rhizosphere microbiome (6.31 ± 0.52 Mb for rhizoplane and 5.87 ± 0.26 Mb for rhizosphere, mean ± SD, *n* = 6, paired *t* test, *p* = 0.01). However, no significant difference in alpha diversity or AGS due to HLB was observed for the rhizosphere and rhizoplane samples. We also performed a variation partitioning analysis to reveal the contribution of the sample layer (rhizosphere/rhizoplane) and health status (healthy/HLB) to the observed taxonomic composition difference between samples. The sample layer was the major determinant of the citrus root-associated microbiome (accounting for 78.87% of the variation observed in the entire microbial community, *p* = 0.024), whereas the contribution of health status was minor (9.32%, *p* = 0.501). The results collectively suggested that the rhizoplane microbiome harbored a distinct, less complex microbial community, in which microorganisms with larger genome size were more abundant than the rhizosphere microbiome. In addition, HLB did not significantly alter the overall structure of the root-associated microbiome (Additional file 1: Figure S2).

Owing to the intimate relationship between microbes and hosts in the rhizoplane, certain microbes are enriched from rhizosphere-to-rhizoplane, as driven by plant selection [5]. The relative abundance of *Proteobacteria* was significantly higher in the rhizoplane samples than the rhizosphere samples, and *Actinobacteria* and *Acidobacteria* were depleted from the rhizosphere to rhizoplane samples (Additional file 1: Figure S3). A more detailed pairwise comparison at the genus level between the rhizosphere and rhizoplane microbial community was performed. Among the 1950 genera identified in all samples, 119 genera exhibited significantly increased relative abundance in the rhizoplane microbiome compared to the rhizosphere microbiome. Among them, *Bradyrhizobium* and *Burkholderia* whose genome sizes are 8.57 ± 0.87 Mb (*n* = 8) and 7.15 ± 0.84 Mb (*n* = 124) based on the available complete genomes in MBGD, respectively (accessed September 13, 2016) [52], were ranked as the top two dominant genera. The average relative abundance of *Bradyrhizobium* and *Burkholderia* were 50.10 ± 5.22% (mean ± SD, *n* = 6) and 10.36 ± 4.60% for the rhizoplane samples and 36.96 ± 6.07% and 5.66 ± 3.99% for the rhizosphere samples*,* respectively. A positive correlation between AGS and the relative abundance of *Bradyrhizobium* was also observed (Spearman’s rank-order correlation, rs = 0.49, *p* < 0.1, *n* = 12). No obvious correlation was observed for *Burkholderia* and AGS, possibly because of its relative low abundance in the samples. Given the large genome size and significantly increased relative abundance in the rhizoplane microbiome compared to the rhizosphere microbiome, we reasoned that *Bradyrhizobium* was the main contributor of the increased AGS observed in the rhizoplane microbiome.

**Fig. 1:**
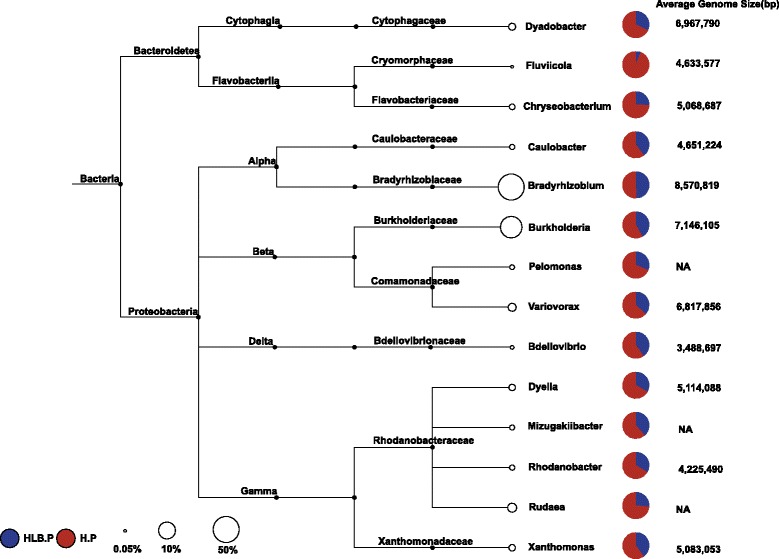
Top rhizoplane-enriched genera with different relative abundances between healthy and HLB rhizoplane samples (*displayed by pie chart*). The average relative abundance of each taxon in the rhizoplane samples is displayed as a percentage. HLB.P (*blue*): rhizoplane samples from HLB trees; H.P (*red*): rhizoplane samples from healthy trees. The genome size for each genus was calculated as the average genome size based on the available complete genomes from MBGD. The full list is shown in Additional file 1: Figure S4

Fifty-two of the 119 rhizoplane-enriched genera exhibited significantly different relative abundance between healthy and HLB rhizoplane samples. Notably, 50 of these 52 genera were more abundant in healthy rhizoplane samples than HLB samples (Fig. 1 and Additional file 1: Figure S4), and the other two genera, *Inquilinus* and *Aureimonas*, contain known human pathogens [53, 54] and exhibited increased relative abundance in HLB rhizoplane samples compared to healthy samples. Among these 50 genera, *Bradyrhizobium* and *Burkholderia* [55, 56] together with several other known root-associated microorganisms such as *Variovorax* [57], *Bdellovibrio* [58], *Chryseobacterium* [59, 60], *Dyadobacter* [61], and *Penicillium* from the fungi kingdom [62] were observed. *Cellvibrio*, which is rhizoplane-enriched and has plant growth-promoting capacity [15, 63], exhibited significantly increased relative abundance in healthy rhizoplane samples compared in HLB samples (Additional file 1: Figure S5A). A nearly complete genome (genome size 5.7 Mb, completeness 99.14% and contamination 0.23% by checkM [43], composing of 41 contigs) representing a novel species of *Cellvibrio* (Additional file 1: Table S5) was generated using the metagenome-assembled genome (MAG) extraction approach [42]. Plant cell wall polysaccharide-degrading enzymes are key features in rhizoplane microbiome [15]. In total, 350 of 4794 genes in the assembled *Cellvibrio* bin were involved in carbohydrate utilization as suggested by dbCAN [64], which was significantly increased compared to the non-plant-associated relatives, such as *Escherichia coli* K12 (132 of 4372 genes are identified, Fisher’s exact test, *p* < 0.0001). Multiple plant growth-promoting-associated genes, such as genes involved in IAA synthesis, were present and actively expressed (Additional file 1: Figure S5B).

Ninety-four rhizoplane-depleted genera were identified, which were mainly affiliated with *Actinobacteria*, *Acidobacteria*, and Archaea, including *Thaumarchaeota*. The majority of rhizoplane-depleted taxa with significantly different relative abundance between healthy and HLB samples (36 of 39 genera) exhibited significantly increased relative abundance in the HLB rhizoplane microbiome compared to healthy samples (Additional file 1: Figure S6). *Thaumarchaea* represents an autotrophic microorganism in the soil microbial community [65]. The relative abundance of *Thaumarchaea* significantly decreased from the rhizosphere to the rhizoplane (*p* < 0.05) and exhibited significantly increased relative abundance in the rhizoplane microbiome of HLB plants compared to healthy trees (Additional file 1: Figure S7A). One high-quality *Thaumarchaeota* MAG was extracted from the metagenome assembly (3.17 Mb, completeness 93.2%, and contamination rate 0.97%). The *amoA* gene-based phylogenetic tree demonstrated that the *Thaumarchaeota* MAG (bin.105) was affiliated with the *Thaumarchaeota* group I.1b (Additional file 1: Figure S7B). The low similarity between bin.105 and the other three available *Thaumarchaeota* group I.1b genomes demonstrated that bin.105 represents a novel member of the *Thaumarchaeota* phylum (Additional file 1: Table S6). Multiple genes involved in the autotrophic life cycle, including nitrification-associated genes (including *amoA* and *amoB*, urease-encoding genes *ure*ABCEFGD) and carbon fixation-associated genes (including 4-hydroxybutyrate-CoA dehydratase, acetyl-CoA carboxylase, and methylmalonyl-CoA), are present in bin.105.

### Effects of HLB on rhizosphere-to-rhizoplane-enriched functional properties

**Fig. 2:**
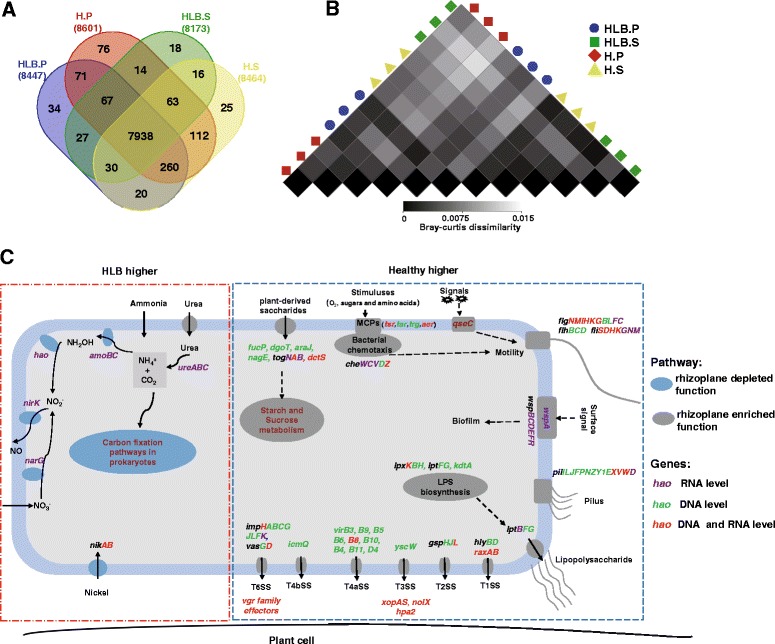
Functional composition of citrus root-associated microbiome. **a** Venn diagram depicting number of KOs identified in rhizosphere and rhizoplane microbiomes from healthy and HLB-diseased samples. **b** Heatmap exhibiting functional composition similarity between samples based on relative abundance data at KO level. **c** Rhizosphere- to rhizoplane-enriched and depleted functions with varying relative abundance and expression levels between healthy and HLB rhizoplane samples. *Dark gray*: rhizoplane-enriched function; *light blue*: rhizoplane-depleted; genes in *green*: increased relative abundance at the DNA level; genes in *purple*: increased expression level; genes in *red*: increased abundance and expression level. HLB.P (*blue*): rhizoplane samples from HLB trees; H.P (*red*): rhizoplane samples from healthy trees; HLB.S (*green*): rhizosphere samples from HLB trees; H.S (*yellow*): rhizosphere samples from healthy trees

Both the KO presence/absence and relative abundance profiling demonstrated that all samples shared very similar functional attributes (Fig. 2a, b). In total, 89.7% of the identified KOs (7938 of 8816) were found in all samples, and the composition similarity among these samples was greater than 98.7%, suggesting that the functional composition of the citrus root-associated microbiome was more conserved than the taxonomic composition (*p* < 2.2E−16) (Fig. 2a, b and Additional file 1: Figure S2). The sample layer contributed significantly to the functional composition variation (80.84%, *p* = 0.036) with a minute contribution from health status (9.57%, *p* = 0.497). We performed the layer KO enrichment analysis to identify the rhizoplane-enriched functional properties. We then determined how HLB affected these rhizoplane-enriched properties at both the genetic potential and gene expression levels. In total, 2218 rhizoplane-enriched KOs were identified. The KOs belonging to the transporter, two-component system, ABC transporter, secretion system, transcription factors, and bacterial motility proteins were the top six enriched functional attributes, highlighting the importance of these functions for microorganisms to adapt to the plant-root surface niche. All identified rhizoplane-enriched KOs were actively expressed, as revealed by the metatranscriptomic data (Additional file 2).

**Fig. 3:**
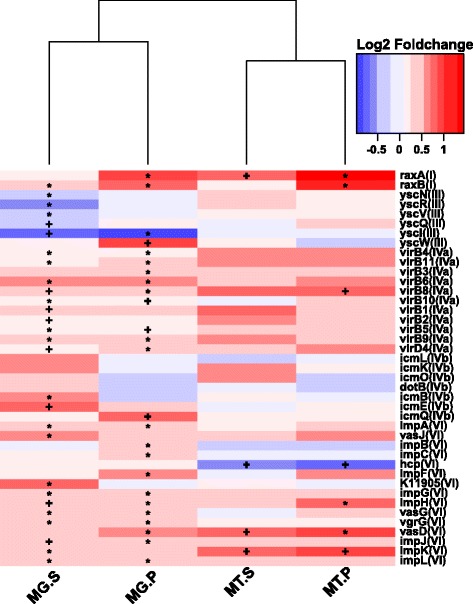
The relative abundance and expression profiling of rhizoplane-enriched genes involved in bacterial secretion systems. Affiliation of each KO with secretion system types (I–VI) is indicated in *brackets* based on KEGG annotation. *Red* denotes “higher in healthy samples,” while blue denotes “higher in HLB samples.” The asterisk denotes *P* < 0.01; the plus sign denotes *P* < 0.05. *MG*, metagenome data, *MT* metatranscriptome data, *P* rhizoplane, *S* rhizosphere

*QseC*, an important quorum-sensing gene controlling bacterial flagella and motility [66], was rhizoplane-enriched and exhibited significantly increased abundance and expression levels in the healthy rhizoplane microbiome compared to the HLB samples. A similar pattern was observed for multiple root surface niche adaption-associated genes, such as bacterial chemotaxis sensor-related genes (including *aer* (aerotaxis (oxygen-sensing) receptor), *tsr* (serine receptor), *tar* (aspartate/maltose receptor), and *trg* (ribose and galactose chemoreceptor)); flagellar assembly-associated genes (Fig. 2c and Additional file 1: Figure S8); the surface contact signal sensing and biofilm formation regulation-associated genes *wspABCDR* [67]; and the chemosensory complex genes involved in pilus synthesis, including *pilILJ*, pilus assembly genes (Fig. 2c and Additional file 1: Figure S9), and LPS synthesis and transporter genes (Fig. 2c and Additional file 1: Figure S10). Multiple genes belonging to the secretion systems, including T1SS (*hlyBD* and *raxAB*), T2SS (*gspCFHIJLM*), T3SS (*yscCJRSTUVNQ*), T4aSS (*virD4* and virB1-11 except *virB7*), and T4bSS (*icmQ*) and T6SS (*ppkA*, *clpV*, *impKL*, *vasD* and *hcp*, and its secreted substrate *vgrG*), were enriched on the rhizoplane. The majority of the identified secretion genes exhibited significantly increased relative abundance and expression activity in healthy rhizoplane samples compared to their HLB counterparts (Figs. 2c and 3). Concomitantly, effector genes, including *xopAS*, *nolX*, and *hpa2*, and several Rhs element *vgr* family genes exhibited significantly increased relative abundance and expression levels in the healthy rhizoplane microbiome compared to HLB samples (Fig. 2c).

**Fig. 4:**
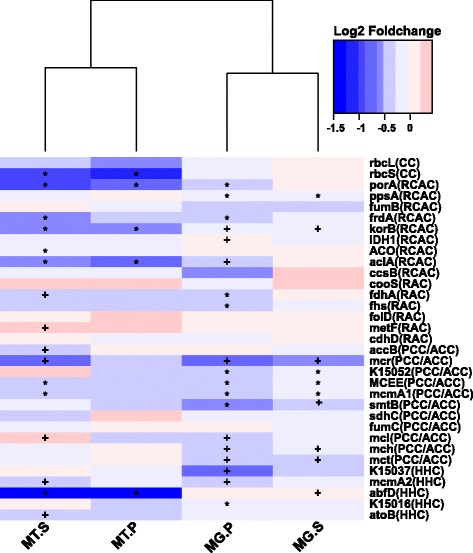
Relative abundance and expression profiling of the rhizoplane-depleted genes involved in carbon fixation. The affiliation of each KO with different carbon fixation pathways is indicated in *brackets* based on KEGG annotation. *CC* Calvin cycle, *RCAC* reductive citric acid cycle, *RAC* reductive acetyl-CoA pathway, *PCC/ACC* 3-hydroxypropionate bicycle, *HHC* hydroxypropionate-hydroxybutyrate cycle. *Red* denotes “higher in healthy samples,” while *blue* denotes “higher in HLB samples.” The asterisk denotes *P* < 0.01; the plus sign denotes *P* < 0.05. *MG* metagenome data, *MT* metatranscriptome data, *P* rhizoplane, *S* rhizosphere

The *yesM-yesN* two-component system, which is involved in carbohydrate utilization for bacteria [68], was enriched from the rhizosphere to rhizoplane microbiome. Several transporter-encoding genes whose products are responsible for importing plant-derived polysaccharide sources to bacterial cells including *togBMNA* (pectin-associated), *fucP* (fucose-associated), *dgoT* (d-galactonate-associated), *araJ* (arabinose-associated), *nagE* (*N*-acetylglucosamine-associated), *lacE* (lactose-associated), and *dctS* (C4-dicarboxylate sensor kinase) were enriched in the rhizoplane microbiome. Furthermore, the relative abundance of all these genes (expect *togM*) was increased in healthy rhizoplane samples. Significantly, increased expression was observed for *togBNA* and *dctS* in the healthy rhizoplane microbiome compared to HLB samples. Consistent with this observation, genes involved in “starch and sucrose metabolism” were enriched in the rhizoplane and exhibited increased abundance and expression levels in the healthy rhizoplane microbiome compared to HLB samples (Fig. 2c and Additional file 1: Figure S11). In contrast, genes involved in “carbon fixation pathways in prokaryotes,” which were depleted in the rhizoplane microbiome, exhibited increased relative abundance and expression activity in HLB rhizoplane samples (Figs. 2c and 4). Increased relative abundance was observed for nitrification-associated key genes (identified as rhizosphere- to rhizoplane-depleted), including *amoA*, *amoB*, *amoC*, and *hao* (hydroxylamine oxidoreductase), in HLB rhizoplane samples compared to healthy samples. The urease-encoding genes *ureA*, *ureB*, and *ureC* involved in degrading urea to ammonia, which contributes to both nitrification and carbon fixation, and the transporter genes for the coenzyme nickel metal for the urease [69], *nikA* and *nikB*, also exhibited significantly increased expression levels in HLB rhizoplane samples compared to healthy samples (Fig. 2c and Additional file 1: Figure S12).

### Linking the rhizoplane-enriched taxonomic and functional properties

**Fig. 5:**
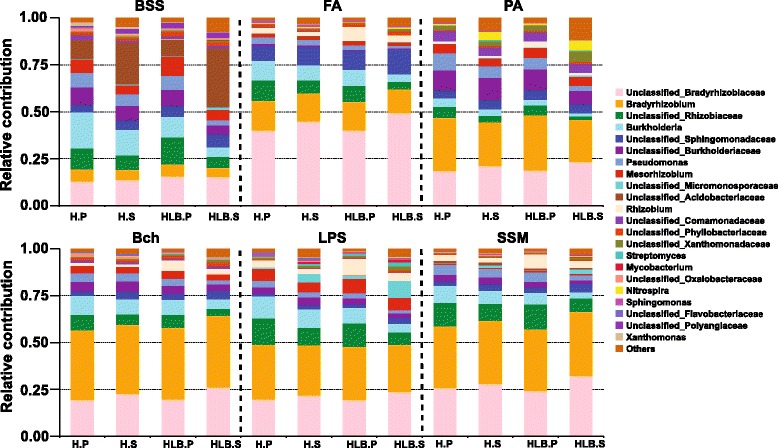
Relative contribution of different taxa (family or genus levels) to identified rhizoplane-enriched functional attributes in different samples. *BSS* bacterial secretion system, *FA* flagellar assembly, *PA* pilus assembly, *BCh* bacteria chemotaxis, *LPS* lipopolysaccharide assembly, *SSM* starch and sucrose metabolism

To visualize the association between rhizoplane-enriched taxonomic and functional properties, we determined the taxonomic origin of rhizoplane-enriched functional attributes for all samples. The rhizoplane-enriched KOs were clustered at KEGG pathway level 3, and the clustered categories involved in host-microbe interactions identified in the above section, including the bacterial secretion system (BSS), flagellar assembly (FA), pilus assembly (PA), bacterial chemotaxis (BCh), and LPS synthesis (LPS), were analyzed, as were the rhizoplane-enriched KOs involved in starch and sucrose metabolism (SSM). *Proteobacteria* were the main contributor of these functions and contributed significantly more to the rhizoplane samples (95.3 ± 0.15% of the normalized total relative contribution for the six functional categories) than the rhizosphere samples (86.6 ± 1.27%) (paired *t* test, *p* < 0.01). A reduced contribution by taxa belonging to *Acidobacteria* and *Actinobacteria* was also observed (two taxa together accounting for 2.98 ± 0.14% of the normalized total relative contribution for the rhizoplane samples and 9.59 ± 1.43% for the rhizosphere samples, paired *t* test, *p* < 0.05) (Fig. 5). The relative contribution of *Bradyrhizobium* and *Burkholderia* to the six functional categories for the rhizoplane samples ranged from 6.2 to 38.1% and 2.9 to 19.2%, respectively, and their relative contribution to the rhizoplane samples significantly increased compared to the rhizosphere samples (*p* < 0.05 for *Bradyrhizobium* and *p* = 3.4E−5 for *Burkholderia*, respectively). The genes involved in the type IVb secretion system identified from our samples were mostly from unclassified bacteria (Additional file 1: Figure S13).

### Expression activity comparison of *Bradyrhizobium* and *Burkholderia* between rhizosphere and rhizoplane samples

*Bradyrhizobium* and *Burkholderia* were the top two dominant and rhizoplane-enriched genera. To reveal the mechanisms underlying their highly efficient colonization, particularly in the rhizoplane, we extracted all genes originating from the two genera from the KO-annotated genes. In all, 2228 *Bradyrhizobium* and 2603 *Burkholderia* KOs were identified. These identified KOs exhibited overall higher relative abundance in the rhizoplane microbiome than the rhizosphere microbiome on a functional potential level (Additional file 1: Figure S14), further demonstrating rhizosphere-to-rhizoplane enrichment for both genera. The differentially expressed (DE) KOs between the rhizoplane and rhizosphere samples for the two genera were further determined using metatranscriptomic data*.* In total, 287 and 185 DE KOs were identified for *Bradyrhizobium* and *Burkholderia*, respectively, including 163 rhizoplane-upregulated and 124 rhizoplane-downregulated KOs for *Bradyrhizobium* and 89 rhizoplane-upregulated and 96 rhizoplane-downregulated KOs for *Burkholderia*. The majority of these rhizoplane-upregulated KOs (153 of 163 for *Bradyrhizobium* and 83 of 89 for *Burkholderia*) were not identified when comparing the rhizoplane and rhizosphere samples at the whole community level using metatranscriptomic data. Overrepresentation of multiple KEGG pathways involved in metabolism was observed for the rhizoplane-downregulated KOs for *Bradyrhizobium* and *Burkholderia*, and consistent enrichment of the categories “energy metabolism” and “carbohydrate metabolism” for both genera was also observed (*p* < 0.05, Fisher’s exact test, two-tail). The *Bradyrhizobium* rhizoplane-upregulated KOs were enriched in the categories of “transcription” and “metabolism|unclassified,” and the *Burkholderia* rhizoplane-upregulated KOs were enriched in the categories of “signal transduction” and “cell motility” (Table 1). All *Burkholderia* rhizoplane-upregulated KOs involved in “cell motility” were responsible for flagellar and pilus assembly; the KO representing the flagellar transcriptional activator *flhD* was found in the “signal transduction” category. Several other efflux system-associated genes, such as *mdtA* and *mdtC*, were also present in the *Burkholderia* “signal transduction” category. The majority of the *Bradyrhizobium* rhizoplane-upregulated KOs belonging to “metabolism|unclassified” were associated with cell wall synthesis. Interestingly, the gene encoding salicylate hydroxylase which converts salicylic acid, a critical phytohormone for plant systemic acquired resistance, to nonfunctional compound catechol was expressed at a significantly increased level in the rhizoplane compared to the rhizosphere for *Bradyrhizobium*.

**Table 1:** Functional distribution of the *Bradyrhizobium* and *Burkholderia* differentially expressed (DE) KOs between rhizosphere and rhizoplane samples

	*Bradyrhizobium*					*Burkholderia*				
Individual functional categories	Background	Rhizoplane downregulated	*p* value	Rhizoplane upregulated	*p* value	Background	Rhizoplane downregulated	*p* value	Rhizoplane upregulated	*p* value
Metabolism of cofactors and vitamins	105	6	0.84	8	0.86	143	1	**0.04**	5	1
Metabolism of other amino acids	51	5	0.40	4	1	55	4	0.28		
Metabolism|unclassified	121	9	0.71	18	*0.04*	148	4	0.66	3	0.49
Metabolism of terpenoids and polyketides	40	2	1	3	1	76	1	0.37	2	1
Energy metabolism	149	31	*5.6E−8*	9	0.29	146	17	*6.6E−5*	3	0.48
Enzyme families	81	3	0.36	7	1	88	3	1	2	0.77
Amino acid metabolism	223	15	1	15	0.31	272	35	*1.4E−10*	6	0.29
Xenobiotics biodegradation and metabolism	97	6	1	10	0.59	108	10	*0.01*	2	0.59
Biosynthesis of other secondary metabolites	19	1	1	3	0.42	23	3	0.07		
Nucleotide metabolism	73	7	0.35	9	0.31	92	2	0.58	5	0.38
Carbohydrate metabolism	195	21	*0.04*	10	0.08	237	17	*0.01*	3	0.06
Lipid metabolism	71	4	1	5	0.83	86	5	0.39	3	1
Glycan biosynthesis and metabolism	41			5	0.42	63	1	0.73	1	0.72
Membrane transport	180	7	0.15	18	0.58	307	13	0.75	15	0.19
Signaling molecules and interaction	3			1	0.29					
Genetic information processing	40	7	*0.03*	1	0.25	69	1	0.52	1	0.52
Folding, sorting, and degradation	51	10	*0.004*	3	0.62	75	3	0.76	4	0.35
Replication and repair	89	8	0.40	8	1	115	1	0.13	6	0.31
Translation	101	4	0.41	11	0.48	138	2	0.17	3	0.48
Signal transduction	121	3	0.08	17	*0.002*	120	4	1	9	*0.04*
Transcription	81	5	1			118	3	0.62	5	0.61
Cellular processes and Ssignaling	128	11	0.47	14	0.42	204	2	**0.03**	11	0.17
Cell growth and death	14			1	1	8			1	0.27
Transport and catabolism	9			1	0.57					
Cell motility						50			7	*0.002*

Background, the functional distribution of *Bradyrhizobium* or *Burkholderia* KOs in all the samples. The KEGG level 2 pathway was used for the functional classification. Rhizoplane-downregulated, the DE KOs show higher expression level in rhizosphere samples than in rhizoplane samples. Rhizoplane-upregulated, the DE KOs show higher expression level in rhizoplane samples than in rhizosphere samples. *p* value, Fisher’s exact test, two-tail. The significant *p* values were italicized, and the underrepresented items were bolded

### Effects of inoculated *Burkholderia* strains on plant fitness

**Fig. 6:**
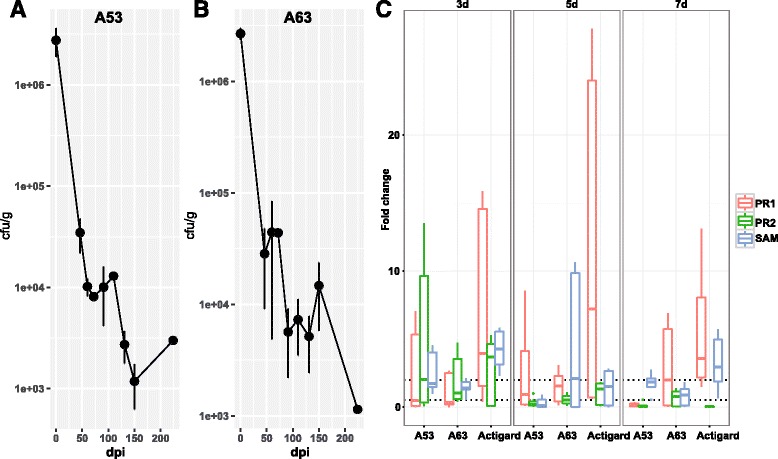
Survival rates of *Burkholderia* spp. inoculation and effects on expression of the citrus ISR-associated genes. Colony-forming units were represented in log10 scale for **a** strain A53 and **b** strain A63. Colonies were counted in NA plates supplemented with kanamycin (50 μg/mL) since both strains are kanamycin-resistant. Days post inoculation (dpi) was calculated up to 225, approximately 7 months post inoculation. *Error bars* represent standard deviation for three biological repeats performed independently. **c** Relative gene expression of three defense-related citrus genes PR1, PR2, and SAM. Fold change of gene expression is represented at 3, 5, and 7 days post inoculation in root system. Fold change was calculated using ∆∆Ct method. *Boxplots* represent fold change compared to non-treated plants for three biological replicates. Each biological replicate contains three technical repeats

Members of *Burkholderia* have been known to benefit plants. Because *Burkholderia* are enriched from the rhizosphere to rhizoplane and it is one of the most abundant bacteria associated with citrus roots, we determined the contribution of *Burkholderia* to the citrus hosts. We isolated multiple *Burkholderia* strains from the rhizosphere of healthy citrus plants in a relevant study (Riera et al., unpublished data). We selected two representative strains A53 (*Burkholderia metallica*) and A63 (*Burkholderia territori*), which showed the best antagonistic activities against *Sinorhizobium meliloti*, a relative of the HLB causal agent Las, and several other citrus pathogens, such as *Phytophthora* spp. and *Alternaria alternate* (Riera et al., unpublished data), to inoculate citrus plants using the soil drench method. The results demonstrated that the two strains successfully colonized the root surface and maintained significantly higher relative abundance inside the rhizoplane bacterial community than inside the rhizosphere bacterial community (Student’s *t* test, *p* < 0.05 for both strains when detected at 5 and 9 dpi) (Additional file 1: Figure S15), demonstrating that the two strains were more adapted to the rhizoplane niche than that to the rhizosphere. The inoculated strains survived well, even 7 months after inoculation (Figs. 6a, b). We then conducted a greenhouse study to evaluate the effects of the selected strains on plant fitness. Salicylic acid (SA)-mediated induced systemic resistance (ISR) is an important benefit of beneficial bacteria to the plant host [70]. We determined the expression of three SA-mediated ISR marker genes, SAM, PR1, and PR2, for the inoculated trees. Plants treated with strain A53 exhibited a significant upregulation of *PR2* gene at 3 dpi compared to negative control plants. A63 induced expression of the SAM gene at 5 dpi and the PR1 gene at 7 dpi. Similarly, Actigard, an analogue of SA, induced PR1 and SAM gene expression at 5 and 7 dpi (Fig. 6c).

## Discussion

Our data demonstrated reduced genetic and microbial complexity in the citrus rhizoplane microbiome compared to that in the rhizosphere communities, indicating the filter effect of plant hosts on the closely associated rhizoplane microbiome assembly [5]. This finding is consistent with previous findings based on annual plants [13, 17], indicating that the rhizosphere-to-rhizoplane enrichment of microbiome occurs in both annual and perennial plants. Our data further suggested that the niche enrichment process is fine-tuned by the plant host for the desired functions, resulting in dramatic structural changes of the microbiome (Fig. 5). Bacteria were the most dominant domain and accounted for more than 99% of the citrus root-associated microbiome, whereas minimal Archaea and fungi were found. The taxonomic composition of the citrus root-associated microbiome identified here was very similar with that of wheat and cucumber, where the relative abundance of bacteria is more than 99% [15]. *Proteobacteria*, which was dominated by *Bradyrhizobium* and *Burkholderia* (Fig. 1), was enriched on the rhizoplane, and depletion of *Actinobacteria* and *Acidobacteria* was observed in this process. We demonstrated that the genes involved in utilizing the root-derived carbon source were enriched from the rhizosphere to the rhizoplane, further suggesting the importance of plant cell wall polysaccharide utilization-associated genes for the rhizoplane microbiome. These genes were gradually enriched from the free living bulk soil inoculum, the rhizosphere to the rhizoplane [15]. However, the genes involved in “carbon fixation pathways in prokaryotes,” which are critical for autotrophic microorganisms, were depleted in rhizoplane samples, collectively highlighting that the plant-derived carbon sources are an important driving force for citrus rhizoplane microbiome assembly. This finding is consistent with the notion that the microbiome on the rhizoplane enjoys superior access to plant exudates compared to the microbiome in the rhizosphere.

Functional features, such as motility, chemotaxis, two-component system and secretion systems, LPS, and type IV pilus synthesis, were rhizoplane-enriched, indicating more active plant-microbe interactions on the rhizoplane than on the rhizosphere. We envision that following chemotaxis sensing, the active motility mediated mainly by flagella and pili allows the microbes to rapidly reach the preferred root surface niches to form biofilms or aggregates with the help of adhesions such as LPS and pili. Interestingly, several secreted effectors were actively expressed in the rhizoplane microbiome that may benefit the microbiome by modulating the plant immune system. Rhizoplane enrichment of *Bradyrhizobium* and *Burkholderia* was observed in this study. Similar enrichment patterns were also observed when analyzing samples collected from 27 important citrus-growing areas worldwide, suggesting the ubiquity and importance of these two genera for the citrus root-associated microbiome (Zhang and Wang, unpublished data). The enrichment of *Bradyrhizobium* and *Burkholderia* on the rhizoplane indicated their successful strategy for rhizoplane adaption. The metatranscriptomic data demonstrated that the functions involved in metabolic activities were more actively expressed in the rhizosphere than the rhizoplane for both genera, further suggesting that nutrient resources were more easily accessed for the rhizoplane microbiome than the rhizosphere microbiome. In addition to common rhizoplane-enriched genes, multiple *Bradyrhizobium* and *Burkholderia* specific genes, such as efflux system-associated genes, cell motility-associated genes, and plant defense-resistant-associated genes, were more active on the rhizoplane than the rhizosphere for *Bradyrhizobium* and *Burkholderia* but were not identified for the remaining microorganisms. The efflux system-associated genes contribute to successful interactions with host plants [71]. The salicylic acid-degrading enzyme salicylate hydroxylase encoding gene from *Bradyrhizobium* exhibited significantly increased expression activity on the rhizoplane and might contribute to the colonization of *Bradyrhizobium* by suppressing the plant defense response [72].

The relative abundance of the rhizoplane-enriched taxa and functional properties, as well as the expression profiling of the rhizoplane-enriched functional features were significantly reduced by HLB. *Bradyrhizobium* and *Burkholderia*, the two most dominant rhizoplane-enriched genera, exhibited reduced relative abundance in HLB rhizoplane samples compared to healthy samples. Our previous cultivation-based results demonstrated that the *Burkholderia* population was significantly increased in the healthy citrus endorhiza microbiome compared to HLB samples, whereas *Bradyrhizobium* was not observed, mainly because of their slow growth (~2 weeks for colony formation. This is longer than the experiment design, during which colonies were harvested after 3–10 days of incubation) [22]. These results suggested that the citrus plants prefer *Bradyrhizobium* and *Burkholderia* and enrich them from the rhizosphere to rhizoplane. The survival rate assay of the two inoculated *Burkholderia* strains further demonstrated that the *Burkholderia* strains were more adapted to the rhizoplane niche than the rhizosphere. Root-associated *Bradyrhizobium* is frequently reported as plant-beneficial bacteria [55, 73]; beneficial effects of *Burkholderia* also are frequently observed [22, 56, 74]. Furthermore, when the citrus were inoculated with *Burkholderia* strains isolated from the healthy citrus root-associated microbiome, several ISR-associated genes in the citrus trees were induced (Fig. 6) that might reduce HLB disease progress. Multiple beneficial bacteria stimulate plant immunity in an SA-dependent ISR manner [70]. The observed upregulation of ISR-associated genes, especially the upregulation of SAM by *Burkholderia* sp. inoculation, likely promotes plant health by triggering SA-dependent broad-spectrum systemic resistance to pathogens, which might be an important aspect of plant-beneficial microbe interactions. Other rhizoplane-enriched taxa, such as *Variovorax* and *Bdellovibrio*, exhibited decreased relative abundance in the rhizoplane microbiome when trees became infected with Las (Fig. 1). *Variovorax* and *Bdellovibrio* exhibit plant growth promotion effects [57, 58]. Additionally, our results revealed rhizoplane-enriched functional properties, including chemotaxis, flagellar assembly, LPS synthesis and transport, as well as secretion system and related effectors, were depleted by HLB. However, the rhizoplane-depleted functional features, including “carbon fixation pathways in prokaryotes” and nitrification and denitrification-related genes, exhibited significantly increased relative abundance and expression levels in the HLB rhizoplane microbiome compared to those in the healthy samples (Figs. 2, 3, and 4 and Additional file 1: Figures S8–S13). Reduced relative abundance and activity of plant-derived polysaccharide utilization-associated genes together with the increased relative abundance and activity of autotrophic life cycle-adapted carbon fixation, nitrification, and denitrification functional features, support our notion that HLB trees supplied less easy-to-use carbon source, e.g., sucrose for root-associated microbiome use [24]. These data are consistent with the notion that HLB affects the availability of sucrose to microbes on the rhizoplane because of its effect on phloem transportation of photoassimilates [18] and root decline [20]. In particular, HLB affects functional features, such as motility, chemotaxis, two-component system and secretion systems, as well as LPS and type IV pilus synthesis with significantly increased relative abundance and expression levels in the healthy rhizoplane microbiome compared in HLB samples (Fig. 2). These findings clearly demonstrated the negative effect on the microbiome at the community level. The negative effects of HLB on the citrus microbiome will ultimately deteriorate the beneficial interactions between the microbiome and the host.

## Conclusions

Overall, we demonstrate that the functional properties involved in host-microbe interactions are critical for the microbiome-inhabiting plant root surfaces and are influenced dramatically by the availability of plant exudates. These rhizoplane-enriched functional properties can subsequently benefit the plant host. HLB not only alters the physiology of the citrus host but also impairs the microbiome-host interaction. Our study provides novel insights for understanding the composition and function of the plant rhizoplane-enriched microbiome and its effect on plant health.

## Additional files

Additional file 1: **Table S1.** Summary of the metagenome data. **Table S2.** Summary of the metagenome assembly and unigenes. **Table S3.** Summary of DNA and RNA reads mapping results. **Table S4.** The alpha diversity (Shannon index) of the microbial communities. **Table S5.** The relationship between the MAG Cellvibrio bin.79 and related genomes. **Table S6.** The relationship between the MAG Thaumarchaeota bin.105 and related genomes. **Figure S1.** The visual symptoms and titer of ‘Ca. L. asiaticus’ in root and leaf samples of HLB symptomatic and healthy citrus trees. **Figure S2.** Taxonomic composition of citrus root-associated microbiome. **Figure S3.** The taxonomic composition of citrus root-associated microbiome. **Figure S4.** The rhizoplane-enriched genera with different relative abundance between healthy and HLB rhizoplane samples. **Figure S5.** (A) The relative abundance of Cellvibrio among all the samples. (B) The expression activity of IAA producing related genes in bin.74. **Figure S6.** The rhizosphere- to rhizoplane-depleted genera with different relative abundance between healthy and HLB rhizoplane samples. **Figure S7.** (A) The relative abundance of Thaumarchaeota among all the samples. (B) The phylogenetic position of the bin.105 based on amoA gene. **Figure S8**-**S12.** The relative abundance and expression profiling of genes involved in flagellar assembly, pilus assembly, LPS assembly, starch and sucrose, and nitrogen metabolism, respectively. **Figure S13.** Relative contribution of different taxa to the identified rhizoplane-enriched genes involved in type IVb secretion system. **Figure S14.** The relative abundance of Bradyrhizobium (A) and Burkholderia (B) KOs among all samples. **Figure S15.** The population dynamic of the inoculated strains in the rhizosphere and rhizoplane bacterial community of the inoculated citrus plants. (DOCX 18086 kb).

Additional file 2: The expression activity of the identified rhizoplane-enriched KOs. Pr denotes the sample is from rhizoplane samples, while Sr denotes the sample is from rhizosphere samples. Samples 1–3 are from healthy trees, and samples 4–6 are from HLB trees. The reads count is shown in the table. (XLSX 233 kb)

## Abbreviations

AGS: Average genome size
CFU: Colony-forming unit
dpi: Days post inoculation
HLB: Huanglongbing
ISR: Induced systemic resistance
KO: KEGG orthologue
MG: Metagenome
MT: Metatranscriptome
SA: Salicylic acid
VPA: Variation partitioning analysis
