# Supplementary material for: Huanglongbing impairs the rhizosphere-to-rhizoplane enrichment process of the citrus root-associated microbiome
Source: Microbiome. 2017 Aug 10;5:97. doi: 10.1186/s40168-017-0304-4 (PMC5553657; doi:10.1186/s40168-017-0304-4)
Supplement: Supplementary file 1 — Table S1. Summary of the metagenome data. Table S2. Summary of the metagenome assembly and unigenes. Table S3. Summary of DNA and RNA reads mapping results. Table S4. The alpha diversity (Shannon index) of the microbial communities. Table S5. The relationship between the MAG Cellvibrio bin.79 and related genomes. Table S6. The relationship between the MAG Thaumarchaeota bin.105 and related genomes. Figure S1. The visual symptoms and titer of ‘Ca. L. asiaticus’ in root and leaf samples of HLB symptomatic and healthy citrus trees. Figure S2. Taxonomic composition of citrus root-associated microbiome. Figure S3. The taxonomic composition of citrus root-associated microbiome. Figure S4. The rhizoplane-enriched genera with different relative abundance between healthy and HLB rhizoplane samples. Figure S5. (A) The relative abundance of Cellvibrio among all the samples. (B) The expression activity of IAA producing related genes in bin.74. Figure S6. The rhizosphere- to rhizoplane-depleted genera with different relative abundance between healthy and HLB rhizoplane samples. Figure S7. (A) The relative abundance of Thaumarchaeota among all the samples. (B) The phylogenetic position of the bin.105 based on amoA gene. Figure S8-S12. The relative abundance and expression profiling of genes involved in flagellar assembly, pilus assembly, LPS assembly, starch and sucrose, and nitrogen metabolism, respectively. Figure S13. Relative contribution of different taxa to the identified rhizoplane-enriched genes involved in type IVb secretion system. Figure S14. The relative abundance of Bradyrhizobium (A) and Burkholderia (B) KOs among all samples. Figure S15. The population dynamic of the inoculated strains in the rhizosphere and rhizoplane bacterial community of the inoculated citrus plants. (DOCX 18086 kb). [file 40168_2017_304_MOESM1_ESM.docx]

Table S1. Summary of the metagenome data

Table S2. Summary of the metagenome assembly and unigenes

Table S3. Summary of DNA and RNA reads mapping results

Table S4. The alpha diversity (Shannon index) of the microbial communities revealed by metagenome data.

Table S5. The relationship between the MAG *Cellvibrio* bin.79 and related genomes.

Table S6. The relationship between the MAG *Thaumarchaeota* bin.105 and related genomes.

Fig. S1. The visual symptoms and titer of ‘*Ca*. L. asiaticus’ in root and leaf samples of HLB symptomatic and healthy citrus trees. Quantification of ‘*Ca*. L. asiaticus’ (genome equivalents per microgram of extracted DNA) by qPCR using primer-probe combination CQULA04FCQULAP10- CQULA04R targeting the beta-operon region of ‘*Ca*. L. asiaticus’. 1-3 were healthy trees and 4-6 were HLB diseased trees.

Fig. S2. Taxonomic composition of citrus root-associated microbiome. (A) Venn diagram depicting number of genera identified in rhizosphere and rhizoplane microbiomes from healthy and HLB diseased samples. (B) Heatmap showing taxonomic composition similarity between samples based on relative abundance data on genus level. (C) PCA plot showing taxonomic composition similarity between samples. HLB.P (blue color): rhizoplane samples from HLB trees; H.P (red color): rhizoplane samples from healthy trees; HLB.S (green color): rhizosphere samples from HLB trees; H.S (yellow color): rhizosphere samples from healthy trees.

Fig. S3. The taxonomic composition of citrus root associated microbiome. The relative abundance of each taxon was calculated based on metagenome reads mapped to the annotated unigenes. Sample 1-3 were from healthy trees while 4-6 were from HLB trees. P denotes rhizoplane sample and S denotes rhizosphere sample.

Fig. S4. The rhizoplane enriched genera with different relative abundance between healthy and HLB rhizoplane samples (displayed by pie chart). The average relative abundance of each taxon in the rhizoplane samples was displayed at the node of each taxon. Blue color, rhizoplane samples from HLB trees; red color, rhizoplane samples from healthy trees.

Figure S5. (A)The relative abundance of *Cellvibrio* among all the samples. This result was based on 16S rDNA OTU data. Approximate 60,000 clean 16S V4 region tags were generated, and the 16S data was analyzed using Uparse pipeline. Sample 1-3 were from healthy trees while 4-6 were from HLB trees. P denotes rhizoplane sample and S denotes rhizosphere sample. (B) The expression activity of IAA producing related genes in bin.74. The metatranscriptome reads were mapped to bin.74 using bowtie2, and the generated alignment files were sorted, indexed using samtools, and viewed using IGV software. The data from healthy tree 2 was shown i.

Fig. S6. The rhizosphere to rhizoplane depleted genera with different relative abundance between healthy and HLB rhizoplane samples (displayed by pie chart). The average relative abundance of each taxon in the rhizoplane samples was displayed at the node of each taxon. Blue color, rhizoplane samples from HLB trees; red color, rhizoplane samples from healthy trees.

Fig. S7. (A) The relative abundance of *Thaumarchaeota* among all the samples. H.P., rhizoplane samples from healthy trees; HLB.S., rhizosphere samples from HLB trees; H.S., rhizosphere samples from healthy trees. (B) The phylogenetic position of the bin.105 based on *amoA* gene.

Fig. S8. The relative abundance and expression profiling of genes involved in flagellar assembly. Red denotes higher in healthy samples while blue denotes higher in HLB samples. *denotes P < 0.01; + denotes P < 0.05. MG, metagenome data. MT, metatranscriptome data. P, rhizoplane. S, rhizosphere.

Fig. S9. The relative abundance and expression profiling of genes involved in pilus assembly. Red denotes higher in healthy samples while blue denotes higher in HLB samples. *denotes P < 0.01; + denotes P < 0.05. MG, metagenome data. MT, metatranscriptome data. P, rhizoplane. S, rhizosphere.

Fig. S10. The relative abundance and expression profiling of genes involved in LPS assembly. Red denotes higher in healthy samples while blue denotes higher in HLB samples. *denotes P < 0.01; + denotes P < 0.05. MG, metagenome data. MT, metatranscriptome data. P, rhizoplane. S, rhizosphere.

Fig. S11. (A) The relative abundance and expression profiling of genes involved in starch and sucrose metabolism. Red denotes higher in healthy samples while blue denotes higher in HLB samples. *denotes P < 0.01; + denotes P < 0.05. MG, metagenome data. MT, metatranscriptome data. P, rhizoplane. S, rhizosphere. (B) Distribution of the identified genes with differential abundance and expression level in rhizoplane samples on the KEGG pathway. The genes which showed higher relative abundance in HLB rhizoplane samples were associated with carbon fixation, while the identified genes with higher abundance and expression level from healthy samples were associated with plant derived carbon source utilization.

Fig. S12. The relative abundance and expression profiling of genes involved in nitrogen metabolism. Red denotes higher in healthy samples while blue denotes higher in HLB samples. *denotes P < 0.01; + denotes P < 0.05. MG, metagenome data. MT, metatranscriptome data. P, rhizoplane. S, rhizosphere.

Fig. S13. Relative contribution of different taxa (on family and genus level) to the identified rhizoplane enriched genes involved in type IVb secretion system.

Fig. S14. The relative abundance of *Bradyrhizobium* (A) and *Burkholderia* (B) KOs among all the 12 samples. The KOs were clustered at KEGG pathway level 3.

Fig. S15. The population dynamic of the inoculated strains in the rhizosphere and rhizoplane bacterial community of the inoculated citrus plants. The relative abundance change compared with the starting point (0dpi, 1 hour after the initial inoculation) of the inoculated strains was calculated using the ∆∆Ct method and the total bacterial population was treated as reference. Red colored, A63 strain; Black colored, A53 strain; solid line, rhizoplane; dash line, rhizosphere. *, the relative abundance in rhizoplane was significantly higher than in rhizosphere (P<0.05); **, P<0.01. Error bar, SE (n=5).

Table S1. Summary of the metagenome data

| Sample | Raw reads | Clean | Sickle_trimmed | Citrus_originated removed (final) | final/clean (%) |
| --- | --- | --- | --- | --- | --- |
| 1Pm | 51,734,540 | 51,306,625 | 49,049,235 | 48,168,809 | 93.88 |
| 1Sm | 46,297,629 | 45,808,357 | 43,541,868 | 43,479,794 | 94.92 |
| 2Pm | 42,775,143 | 42,392,528 | 40,421,462 | 40,055,489 | 94.49 |
| 2Sm | 44,717,963 | 44,278,712 | 42,138,671 | 41,964,779 | 94.77 |
| 3Pm | 46,887,762 | 46,354,018 | 43,673,263 | 42,979,496 | 92.72 |
| 3Sm | 50,094,983 | 49,594,322 | 47,014,547 | 46,784,186 | 94.33 |
| 4Pm | 46,493,708 | 42,274,227 | 40,449,070 | 40,260,462 | 95.24 |
| 4Sm | 44,765,457 | 40,684,234 | 38,890,353 | 38,875,121 | 95.55 |
| 5Pm | 44,502,053 | 44,036,676 | 41,779,557 | 41,499,390 | 94.24 |
| 5Sm | 43,170,586 | 42,687,905 | 40,400,003 | 40,363,893 | 94.56 |
| 6Pm | 39,658,429 | 39,291,988 | 37,416,839 | 37,016,694 | 94.21 |
| 6Sm | 41,983,150 | 41,613,525 | 39,840,087 | 39,723,514 | 95.46 |

Notes: Pm denotes the sample was from rhizoplane, and Sm denotes the sample was from rhizosphere. Sample 1-3 were from healthy trees while 4-6 were from HLB trees. The clean reads were further trimmed using Sickle with parameters -l 80 and q 20. Citrus_originated removed: the reads were mapped to sweet orange, *C. clementina* and Swingle citrumelo genome and the unmapped pair end reads were kept.

Table S2. Summary of the metagenome assembly and unigenes

|  | Final contigs (>=200 bp) | Unigene (0.95 identity) |
| --- | --- | --- |
| # sequence | 17,676,569 | 21,380,400 |
| Total length (bp) | 10,843,633,101 | 9,062,540,994 |
| Longest length (bp) | 536,098 | 27,087 |
| N50 (bp) | 651 | / |
| GC (%) | 62.72 | 64.43 |
| DNA aligned rate (%) | 49.69 | 40.96 |
| RNA aligned rate (%) / | | 30.16 |

Table S3. Summary of DNA and RNA reads mapping results

| Sample ID | Alignment rate (DNA to assembly, %) | Alignment rate (DNA to unigene, %) | Alignment rate (RNA to unigene, %) | Aligned Gene Number (DNA) | Aligned Gene Number (RNA) | Active rate (RNA/DNA, %) |
| --- | --- | --- | --- | --- | --- | --- |
| 1P | 52.42 | 39.98 | 22.76 | 8,017,764 | 2,097,047 | 19.36 |
| 2P | 57.77 | 49.92 | 33.75 | 6,409,407 | 3,444,215 | 41.14 |
| 3P | 59.01 | 52.98 | 44.39 | 7,532,101 | 2,491,298 | 25.41 |
| 4P | 54.05 | 47.25 | 36.53 | 8,172,598 | 3,476,926 | 32.49 |
| 5P | 53.81 | 41.37 | 23.57 | 7,234,026 | 1,890,775 | 19.76 |
| 6P | 54.41 | 47.4 | 36.62 | 7,732,205 | 2,340,609 | 21.92 |
| 1S | 41.66 | 34.01 | 26.01 | 8,141,138 | 2,282,347 | 19.57 |
| 2S | 48.62 | 39.79 | 30.44 | 7,503,003 | 2,529,895 | 24.74 |
| 3S | 41.72 | 32.92 | 31.8 | 8,185,646 | 3,453,416 | 30.77 |
| 4S | 44.26 | 35.08 | 25.74 | 7,782,293 | 2,222,661 | 19.56 |
| 5S | 44.19 | 35.59 | 18.94 | 6,315,165 | 1,703,694 | 18.45 |
| 6S | 44.3 | 35.25 | 31.36 | 7,888,771 | 2,005,419 | 18.01 |

Note: Sample 1-3 were from healthy trees while 4-6 were from HLB trees. P denotes rhizoplane sample and S denotes rhizosphere sample.

Table S4. The alpha diversity (Shannon index) of the microbial communities revealed by metagenome data.

| Sample Name | Phylum | Class | Order | Family | Genus |
| --- | --- | --- | --- | --- | --- |
| 1Pm | 0.715 | 1.274 | 1.606 | 2.140 | 2.485 |
| 1Sm | 0.909 | 1.492 | 2.066 | 2.567 | 3.293 |
| 2Pm | 0.749 | 1.400 | 1.659 | 2.006 | 2.121 |
| 2Sm | 1.040 | 1.640 | 2.172 | 2.543 | 3.045 |
| 3Pm | 0.579 | 1.302 | 1.560 | 2.031 | 2.125 |
| 3Sm | 1.101 | 1.670 | 2.341 | 2.880 | 3.567 |
| 4Pm | 0.754 | 1.291 | 1.585 | 2.071 | 2.287 |
| 4Sm | 1.109 | 1.648 | 2.424 | 2.940 | 3.843 |
| 5Pm | 0.801 | 1.417 | 1.837 | 2.394 | 2.910 |
| 5Sm | 1.039 | 1.752 | 2.467 | 3.124 | 4.198 |
| 6Pm | 0.692 | 1.293 | 1.606 | 2.077 | 2.304 |
| 6Sm | 1.138 | 1.645 | 2.379 | 2.873 | 3.724 |
| t test (S vs. P, two tail) | 2.17579E-05 | 2.54E-05 | 5.21E-06 | 7.34E-05 | 0.000129 |

Table S5. The relationship between the MAG *Cellvibrio* bin.79 and related genomes

| **Query genome** | **Reference genome** | **DDH** | **Model C.I.** | **Bootstrap C.I.** | **Distance** | **Prob. DDH >= 70%** |
| --- | --- | --- | --- | --- | --- | --- |
| **bin.79** | ***Cellvibrio japonicus* Ueda107** | **19.8** | **[17.6 - 22.3%]** | **19.8 - 19.9%** | **0.2215** | **0** |
| **bin.79** | ***Cellvibrio* sp. BR** | **19.6** | **[17.4 - 22%]** | **19.6 - 19.6%** | **0.2238** | **0** |
| **bin.79** | ***Cellvibrio mixtus* ssp. mixtus J3-8** | **24.6** | **[22.2 - 27%]** | **24.5 - 24.6%** | **0.1776** | **0.01** |
| **bin.79** | ***Cellvibrio* sp. OA-2007** | **19.4** | **[17.2 - 21.8%]** | **19.4 - 19.4%** | **0.2268** | **0** |
| **bin.79** | ***Cellvibrio* sp. pealriver** | **20.6** | **[18.4 - 23%]** | **20.6 - 20.6%** | **0.2135** | **0** |

Table S6. The relationship between the MAG *Thaumarchaeota* bin 105 and related genomes

| **Query genome** | **Reference genome** | **DDH** | **Model C.I.** | **Bootstrap C.I.** | **Distance** | **Prob. DDH >= 70%** |
| --- | --- | --- | --- | --- | --- | --- |
| **bin.105** | ***Nitrososphaera gargensis* Ga9.2** | **22.7** | **[20.5 - 25.2%]** | **22.6 – 22.8%** | **0.1927** | **0** |
| **bin.105** | ***Nitrososphaera viennensis* EN76** | **17.5** | **[15.4 - 19.9%]** | **17.4 - 17.7%** | **0.2506** | **0** |
| **bin.105** | ***Thaumarchaeota archaeon* MY3** | **23.1** | **[20.8 - 25.6%]** | **23 - 23.2%** | **0.1894** | **0** |


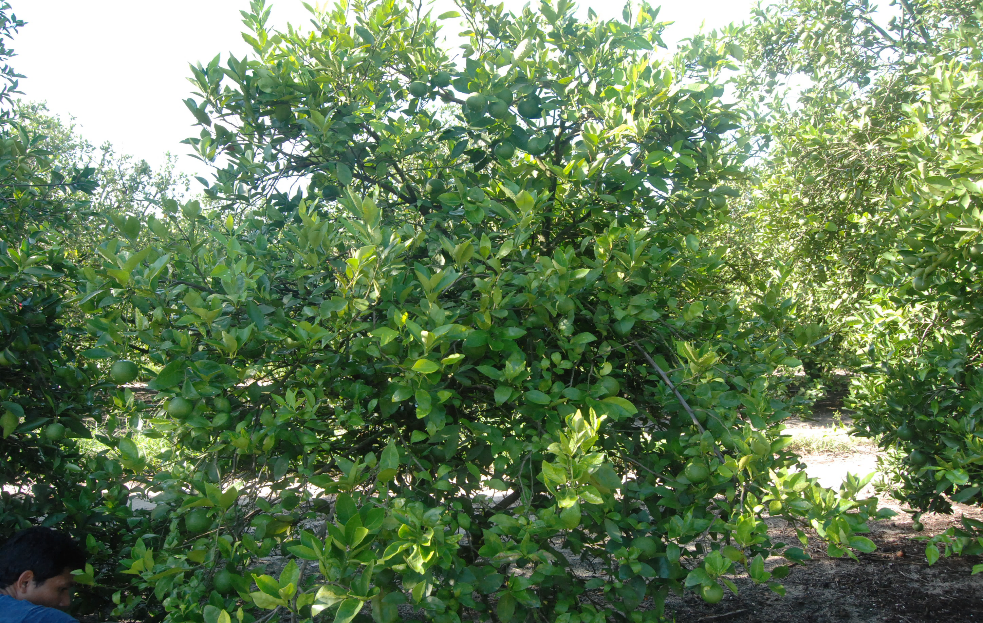

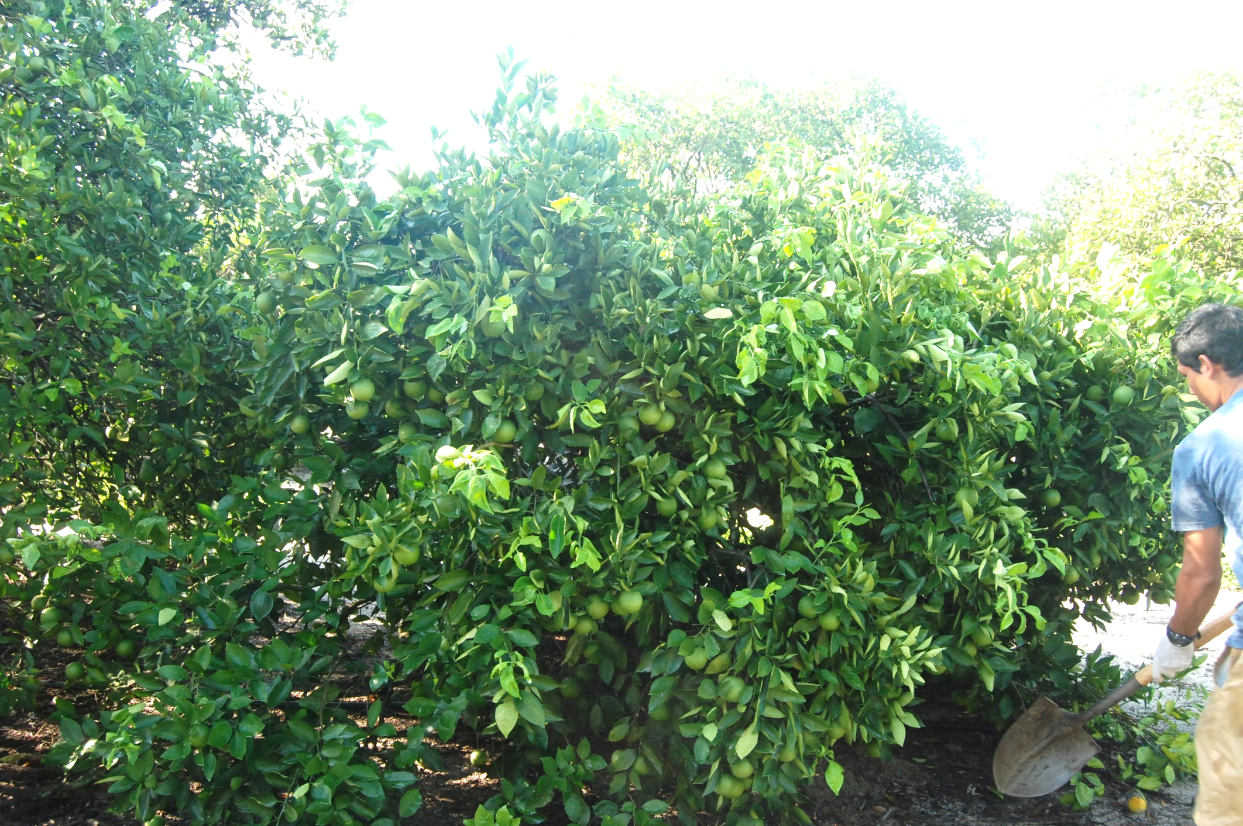


HLB

Healthy


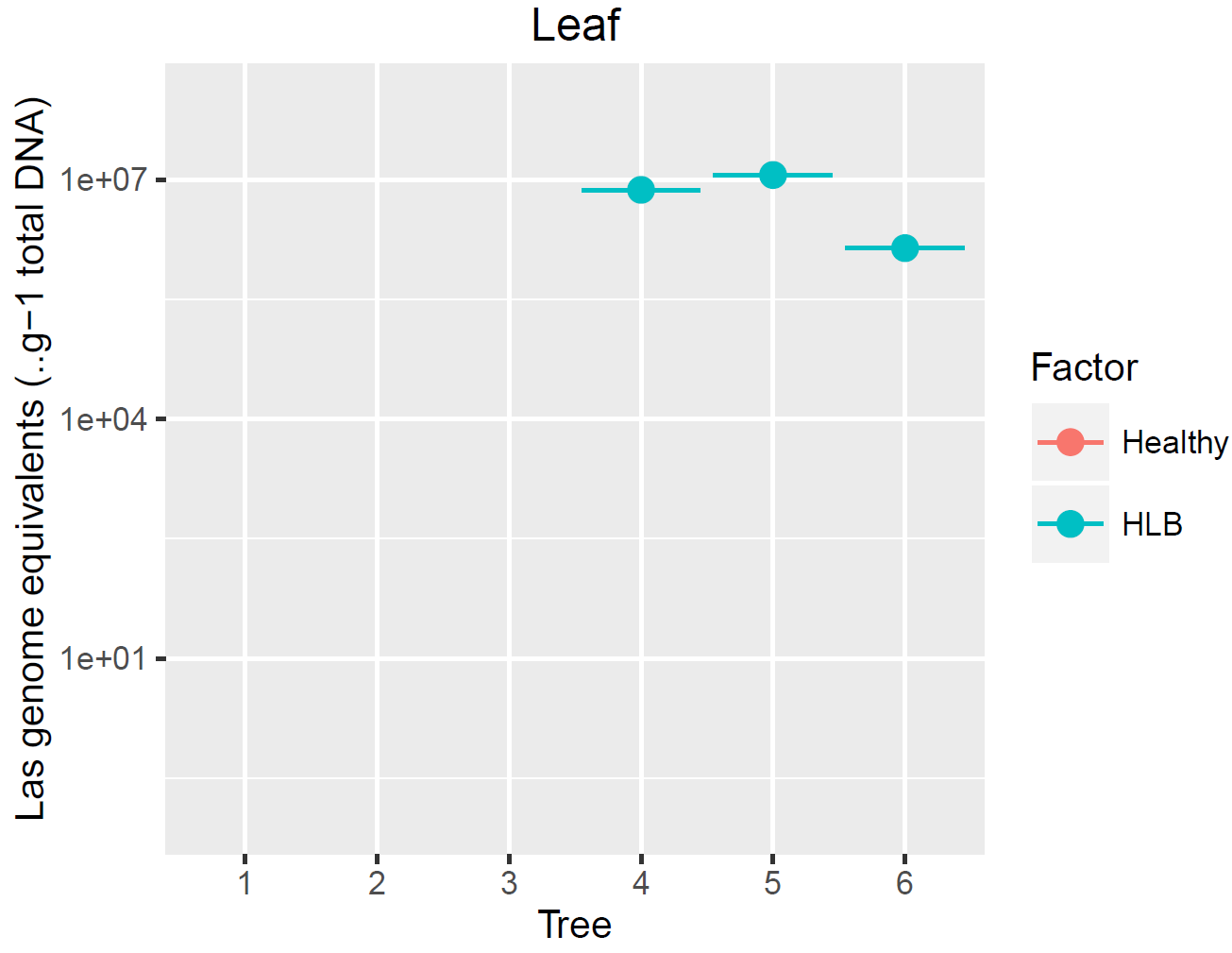

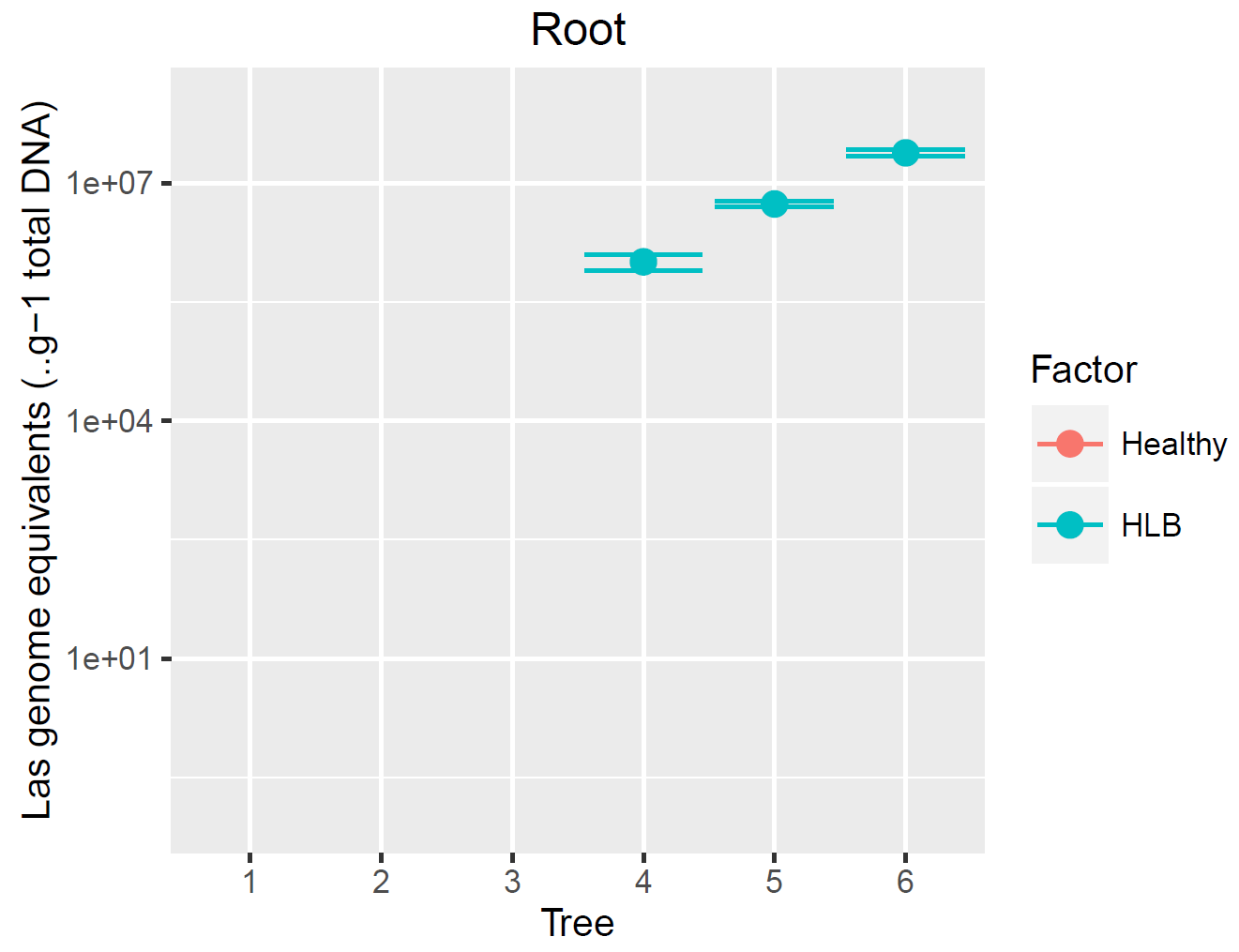


Fig. S1. The visual symptoms and titer of ‘*Ca*. L. asiaticus’ in root and leaf samples of HLB symptomatic and healthy citrus trees. Quantification of ‘*Ca*. L. asiaticus’ (genome equivalents per microgram of extracted DNA) by qPCR using primer-probe combination CQULA04FCQULAP10- CQULA04R targeting the beta-operon region of ‘*Ca*. L. asiaticus’. 1-3 were healthy trees and 4-6 were HLB diseased trees.


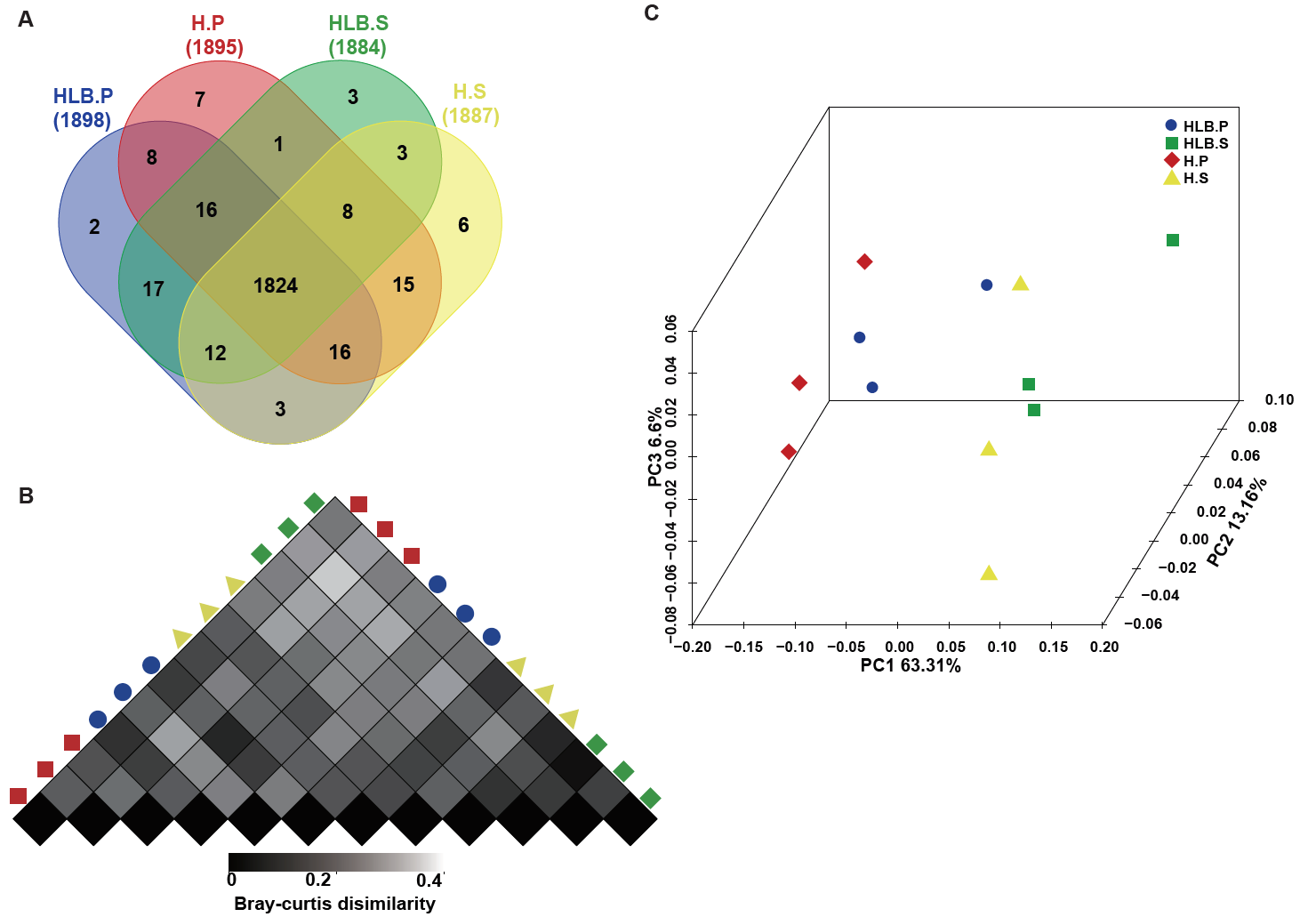


Fig. S2. Taxonomic composition of citrus root-associated microbiome. (A) Venn diagram depicting number of genera identified in rhizosphere and rhizoplane microbiomes from healthy and HLB diseased samples. (B) Heatmap showing taxonomic composition similarity between samples based on relative abundance data on genus level. (C) PCA plot showing taxonomic composition similarity between samples. HLB.P (blue color): rhizoplane samples from HLB trees; H.P (red color): rhizoplane samples from healthy trees; HLB.S (green color): rhizosphere samples from HLB trees; H.S (yellow color): rhizosphere samples from healthy trees.


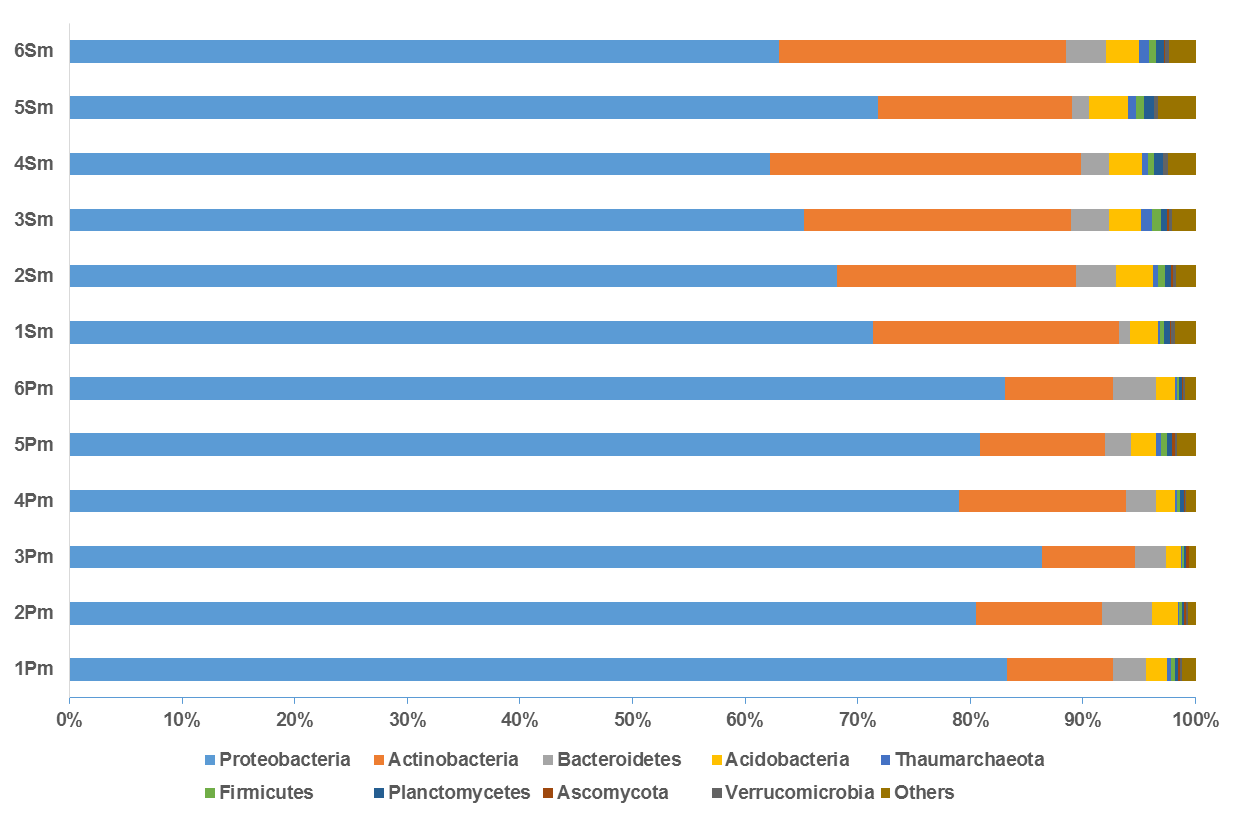


Fig. S3. The taxonomic composition of citrus root associated microbiome. The relative abundance of each taxon was calculated based on metagenome reads mapped to the annotated unigenes. Sample 1-3 were from healthy trees while 4-6 were from HLB trees. P denotes rhizoplane sample and S denotes rhizosphere sample.


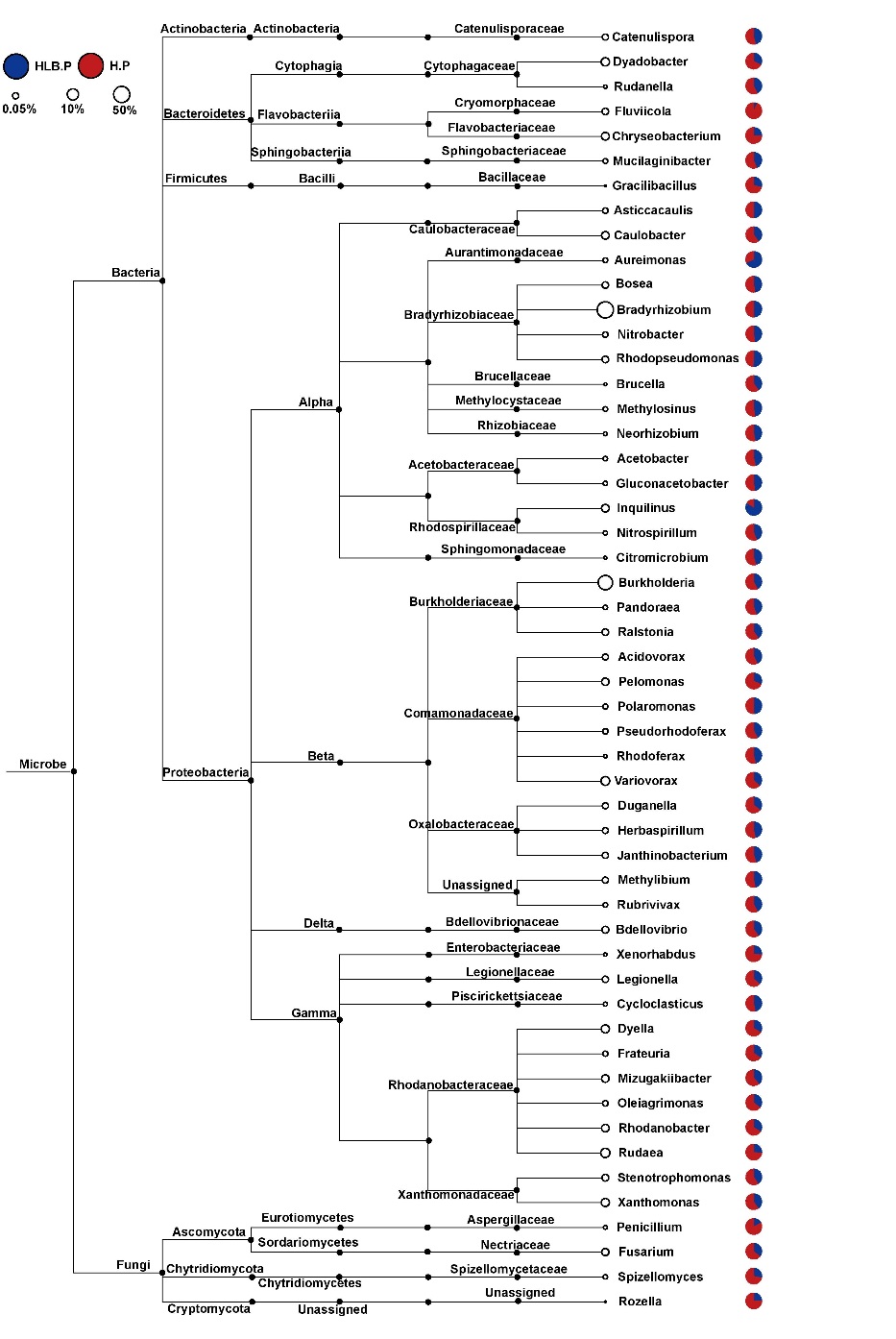


Fig. S4. The rhizoplane enriched genera with different relative abundance between healthy and HLB rhizoplane samples (displayed by pie chart). The average relative abundance of each taxon in the rhizoplane samples was displayed at the node of each taxon. Blue color, rhizoplane samples from HLB trees; red color, rhizoplane samples from healthy trees.


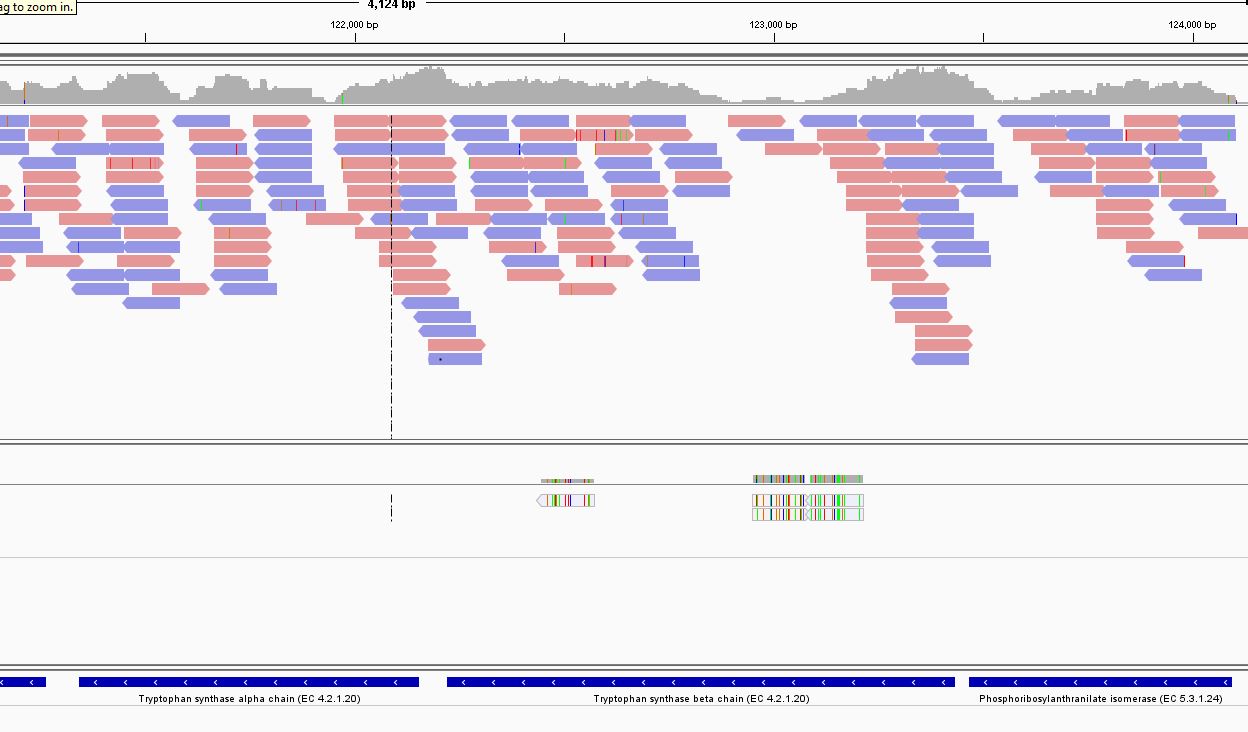


**B**

**A**


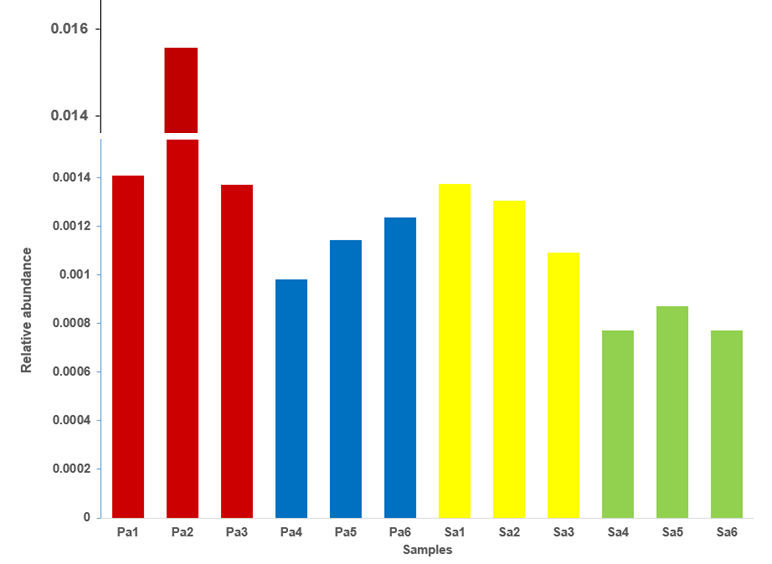


*** *P*<0.05**

*** *P*<0.05**

**H.P**

**HLB.P**

**H.S**

**HLB.S**

Figure S5. (A)The relative abundance of *Cellvibrio* among all the samples. This result was based on 16S rDNA OTU data. Approximate 60,000 clean 16S V4 region tags were generated, and the 16S data was analyzed using Uparse pipeline. Sample 1-3 were from healthy trees while 4-6 were from HLB trees. P denotes rhizoplane sample and S denotes rhizosphere sample. (B) The expression activity of IAA producing related genes in bin.74. The metatranscriptome reads were mapped to bin.74 using bowtie2, and the generated alignment files were sorted, indexed using samtools, and viewed using IGV software. The data from healthy tree 2 was shown here as an example.


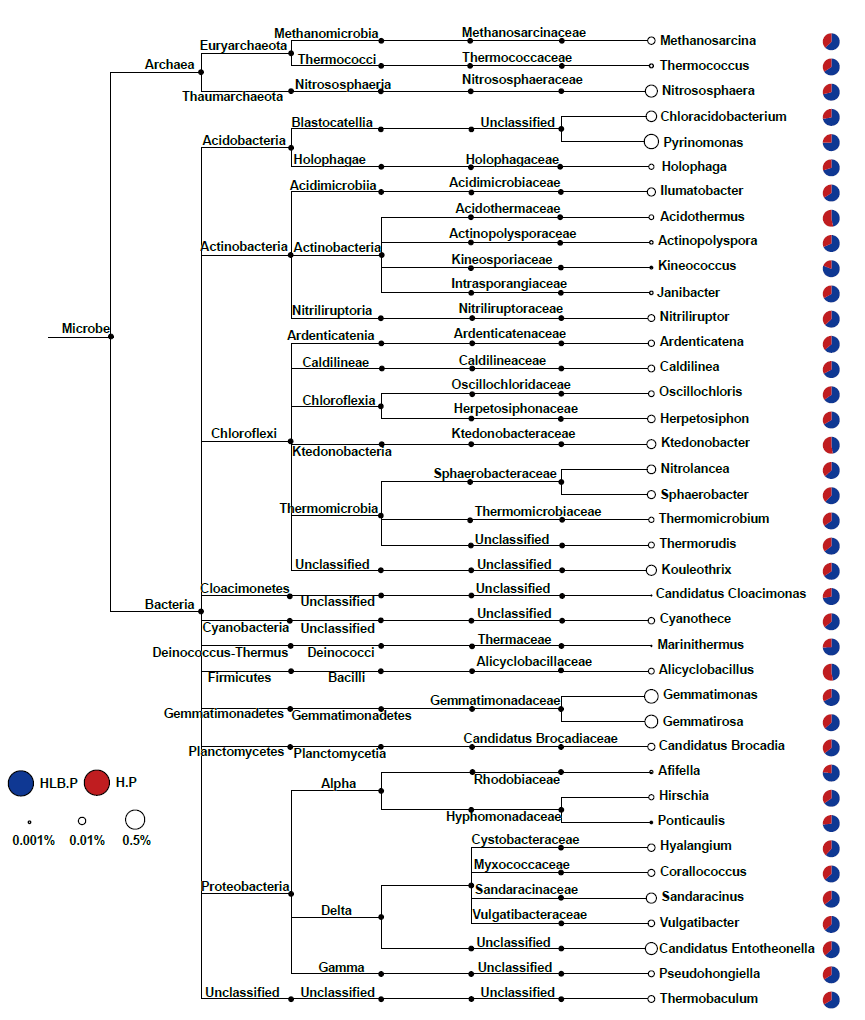


Fig. S6. The rhizosphere to rhizoplane depleted genera with different relative abundance between healthy and HLB rhizoplane samples (displayed by pie chart). The average relative abundance of each taxon in the rhizoplane samples was displayed at the node of each taxon. Blue color, rhizoplane samples from HLB trees; red color, rhizoplane samples from healthy trees.

Figure S7. (A) The relative abundance of *Thaumarchaeota* among all the samples. H.P., rhizoplane samples from healthy trees; HLB.S., rhizosphere samples from HLB trees; H.S., rhizosphere samples from healthy trees. (B) The phylogenetic position of the bin.105 based on *amoA* gene.


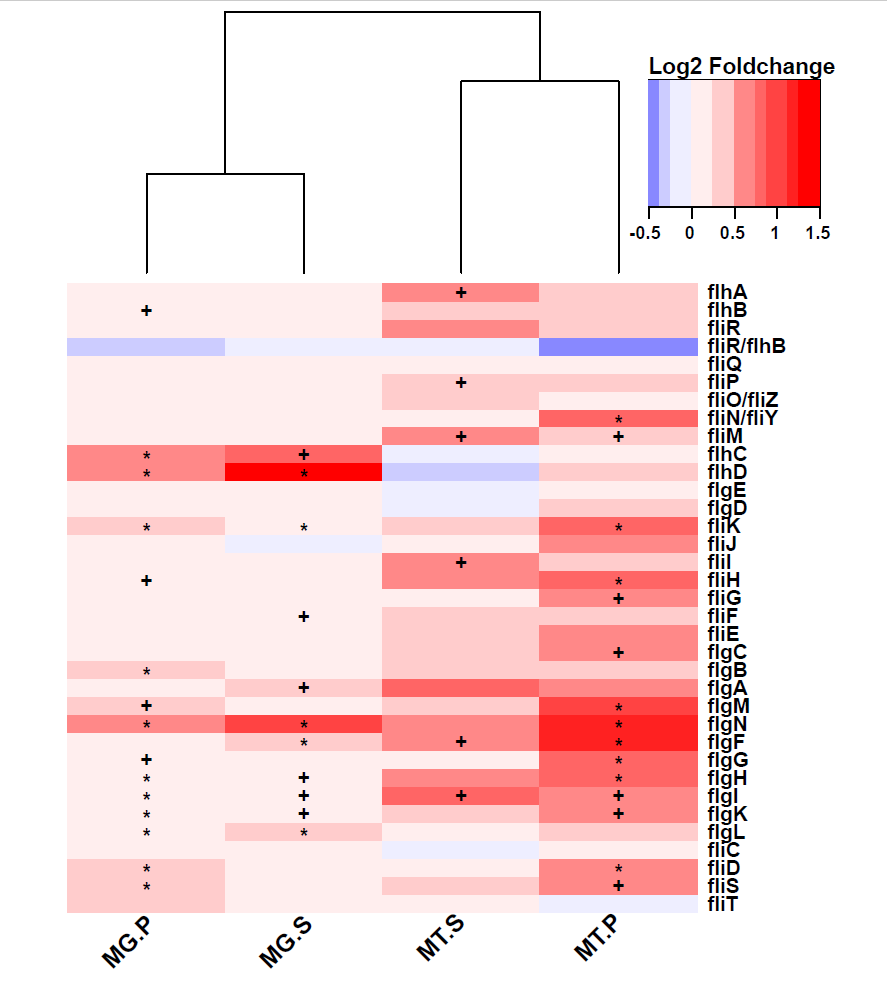


Fig. S8. The relative abundance and expression profiling of genes involved in flagellar assembly. Red denotes higher in healthy samples while blue denotes higher in HLB samples. *denotes P < 0.01; + denotes P < 0.05. MG, metagenome data. MT, metatranscriptome data. P, rhizoplane. S, rhizosphere.


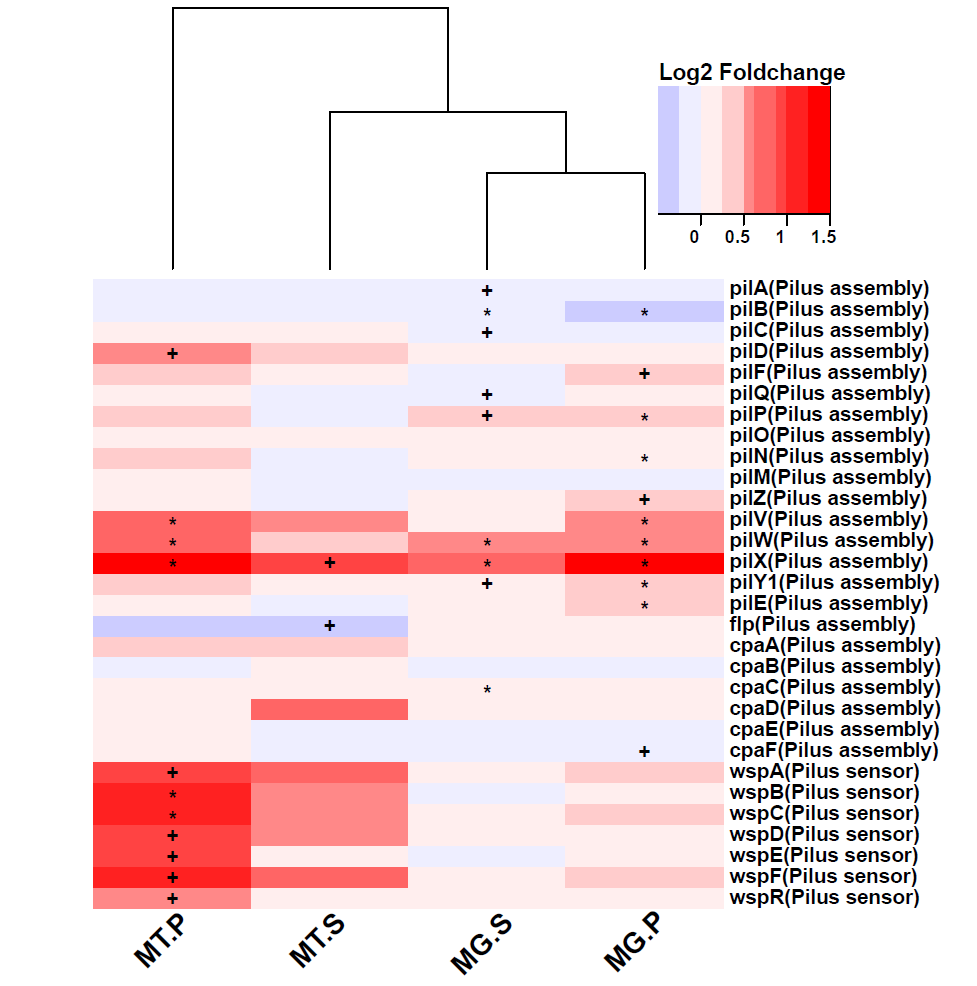


Fig. S9. The relative abundance and expression profiling of genes involved in pilus assembly. Red denotes higher in healthy samples while blue denotes higher in HLB samples. *denotes P < 0.01; + denotes P < 0.05. MG, metagenome data. MT, metatranscriptome data. P, rhizoplane. S, rhizosphere.


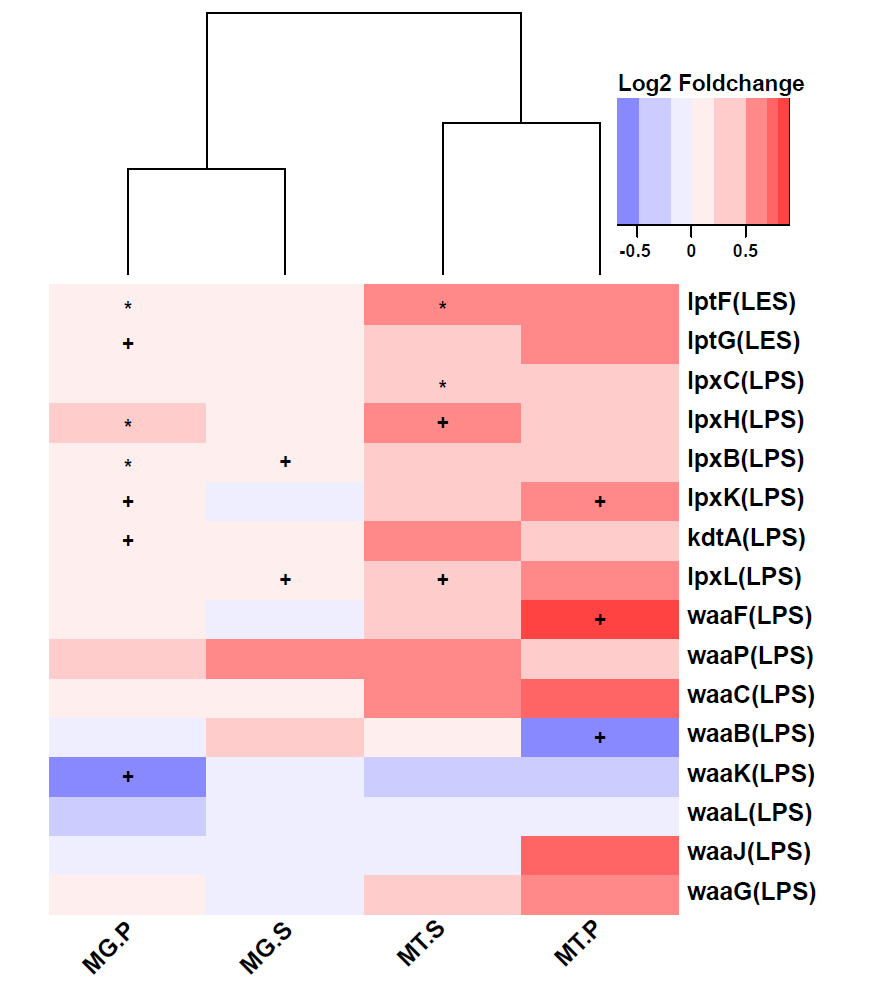


Fig. S10. The relative abundance and expression profiling of genes involved in LPS assembly. Red denotes higher in healthy samples while blue denotes higher in HLB samples. *denotes P < 0.01; + denotes P < 0.05. MG, metagenome data. MT, metatranscriptome data. P, rhizoplane. S, rhizosphere.

Fig. S11. (A) The relative abundance and expression profiling of genes involved in starch and sucrose metabolism. Red denotes higher in healthy samples while blue denotes higher in HLB samples. *denotes P < 0.01; + denotes P < 0.05. MG, metagenome data. MT, metatranscriptome data. P, rhizoplane. S, rhizosphere. (B) Distribution of the identified genes with differential abundance and expression level in rhizoplane samples on the KEGG pathway. The genes which showed higher relative abundance in HLB rhizoplane samples were associated with carbon fixation, while the identified genes with higher abundance and expression level from healthy samples were associated with plant derived carbon source utilization.


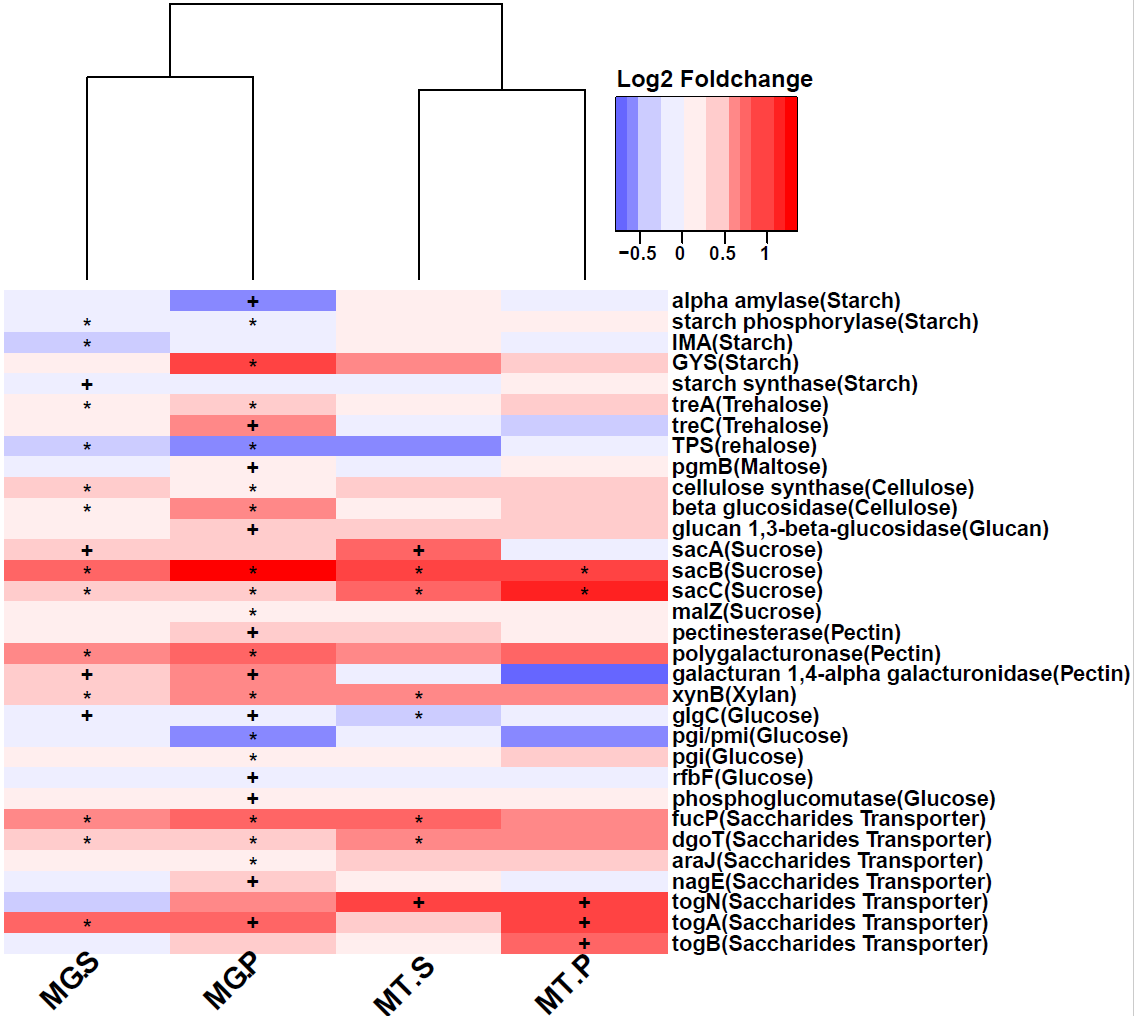


A

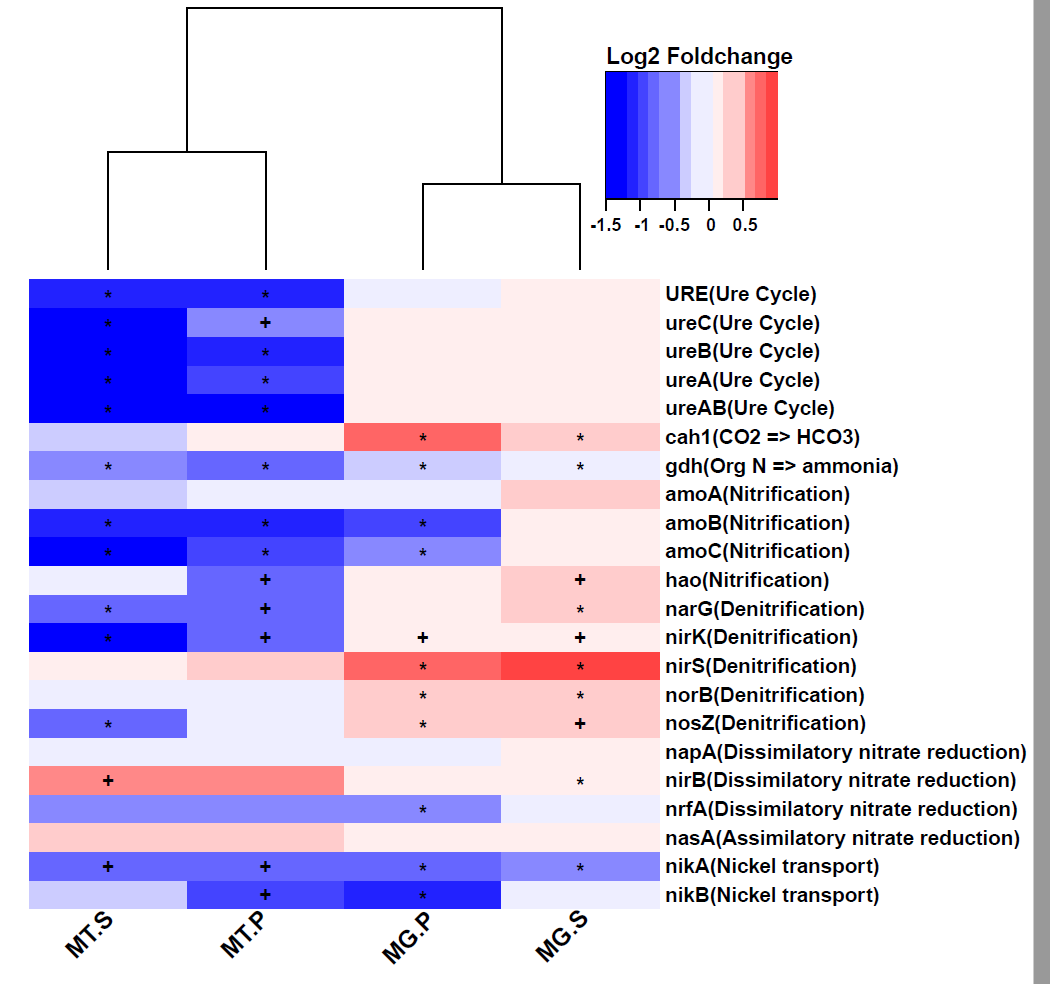


Fig. S12. The relative abundance and expression profiling of genes involved in nitrogen metabolism. Red denotes higher in healthy samples while blue denotes higher in HLB samples. *denotes P < 0.01; + denotes P < 0.05. MG, metagenome data. MT, metatranscriptome data. P, rhizoplane. S, rhizosphere.


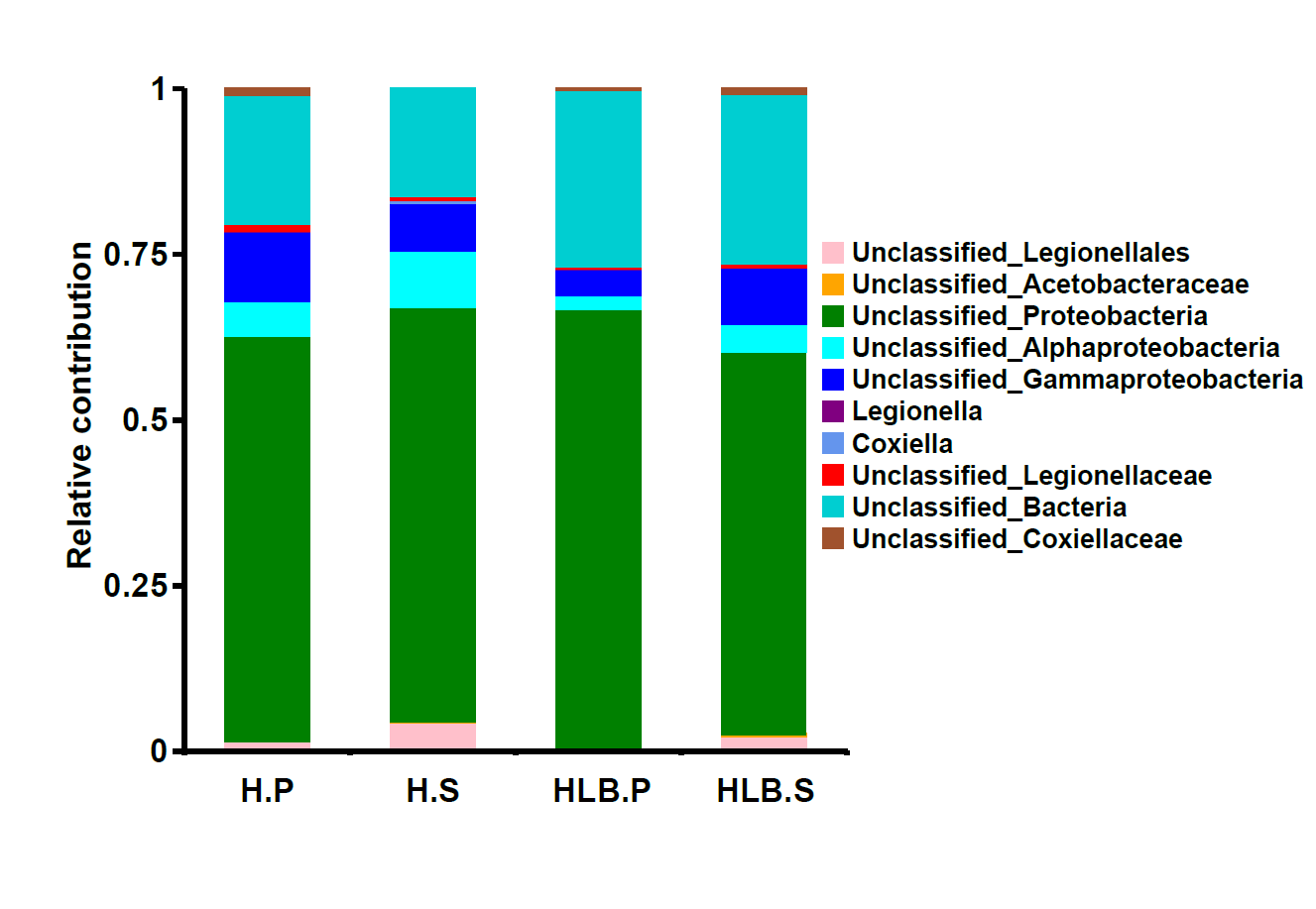


Fig. S13. Relative contribution of different taxa (on family and genus level) to the identified rhizoplane enriched genes involved in type IVb secretion system.


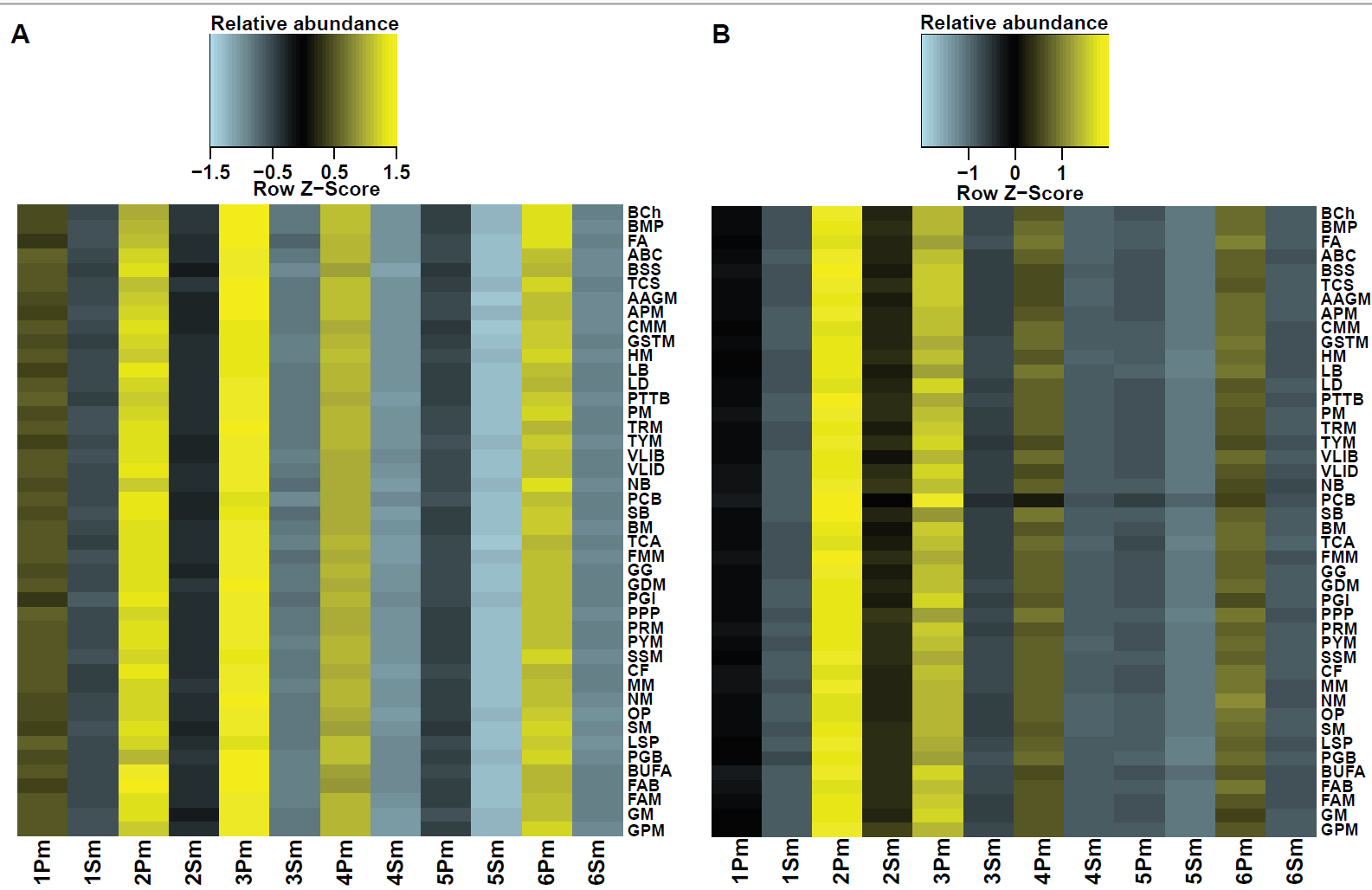


Fig. S14. The relative abundance of *Bradyrhizobium* (A) and *Burkholderia* (B) KOs among all the 12 samples. The KOs were clustered at KEGG pathway level 3.

Fig. S15. The population dynamic of the inoculated strains in the rhizosphere and rhizoplane bacterial community of the inoculated citrus plants. The relative abundance change compared with the starting point (0dpi, 1 hour after the initial inoculation) of the inoculated strains was calculated using the ∆∆Ct method and the total bacterial population was used as a reference. Red color: A63 strain; Black color: A53 strain; solid line: rhizoplane; dash line: rhizosphere. *, the relative abundance in rhizoplane was significantly higher than in rhizosphere (P<0.05); **, P<0.01. Error bar, SE (n=5).
